# Supplementary material for: Engaging Precision Phenotyping to Scrutinize Vegetative Drought Tolerance and Recovery in Chickpea Plant Genetic Resources
Source: Plants (Basel). 2023 Aug 4;12(15):2866. doi: 10.3390/plants12152866 (PMC10421427; doi:10.3390/plants12152866)

# Figure S

## Contents

Figure S ........ 1

Figure S1: Heritability of dry and fresh weight from DAT 43........ 2

Figure S2: Heritability of image-derived traits. ........ 3

Figure S3: Heritability of Chlorophyll Fluorescence traits ........ 4

Figure S4: Correlation of EB from DAT 42 and fresh weight from DAT 43 ........ 5

Figure S5: Relative growth rate of drought stress treatment. ........ 6

Figure S6: Coefficient of variation of image-derived traits ........ 7

Figure S7: Chlorophyll Fluorescence traits for drought phases ........ 8

Figure S8: Correlation among chlorophyll fluorescence traits........ 9

Figure S9: Correlation of ΦPSIIh and image-derived traits for DAT 27. ........ 10

Figure S10: Correlation matrix of Chlorophyll Fluorescence and image-derived traits ........ 11

Figure S11: PCA for desi and kabuli for DT. ........ 12

Figure S13: Loss of drought stress compared to control treatment of *desi* and *kabuli* ........ 14

Figure S 14: Loss of drought stress compared to control treatment of biological status........ 15

Figure S 15: Deviation of the individual genotypes with their affiliation to the biological status, from the mean value of EB of all 60 genotypes. ........ 16

Figure S 16: WUE of *desi* and *kabuli* during drought phases ........ 17

Figure S 17: Deviation of the individual genotypes with their affiliation to *desi* and *kabuli*, from the mean value of all 60 genotypes. ........ 18

Figure S 18: Deviation of the individual genotypes from the mean value of ϕ PS IIh of all 60 genotypes........ 19

Figure S1: Heritability of dry and fresh weight from DAT 43.

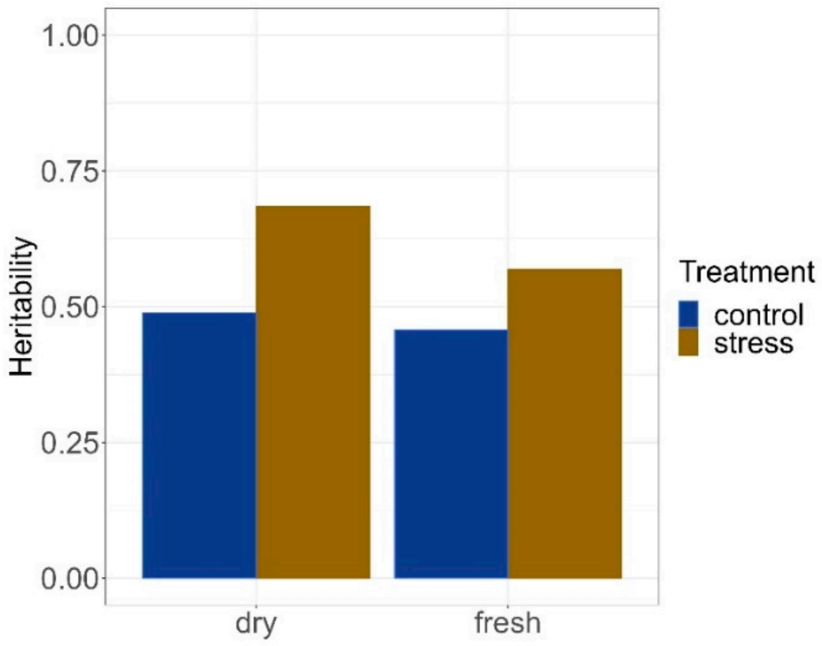

Figure S2: Heritability of image-derived traits.

A.) Plant Height; B.) MCV; C.) r2g; D.) y2g.

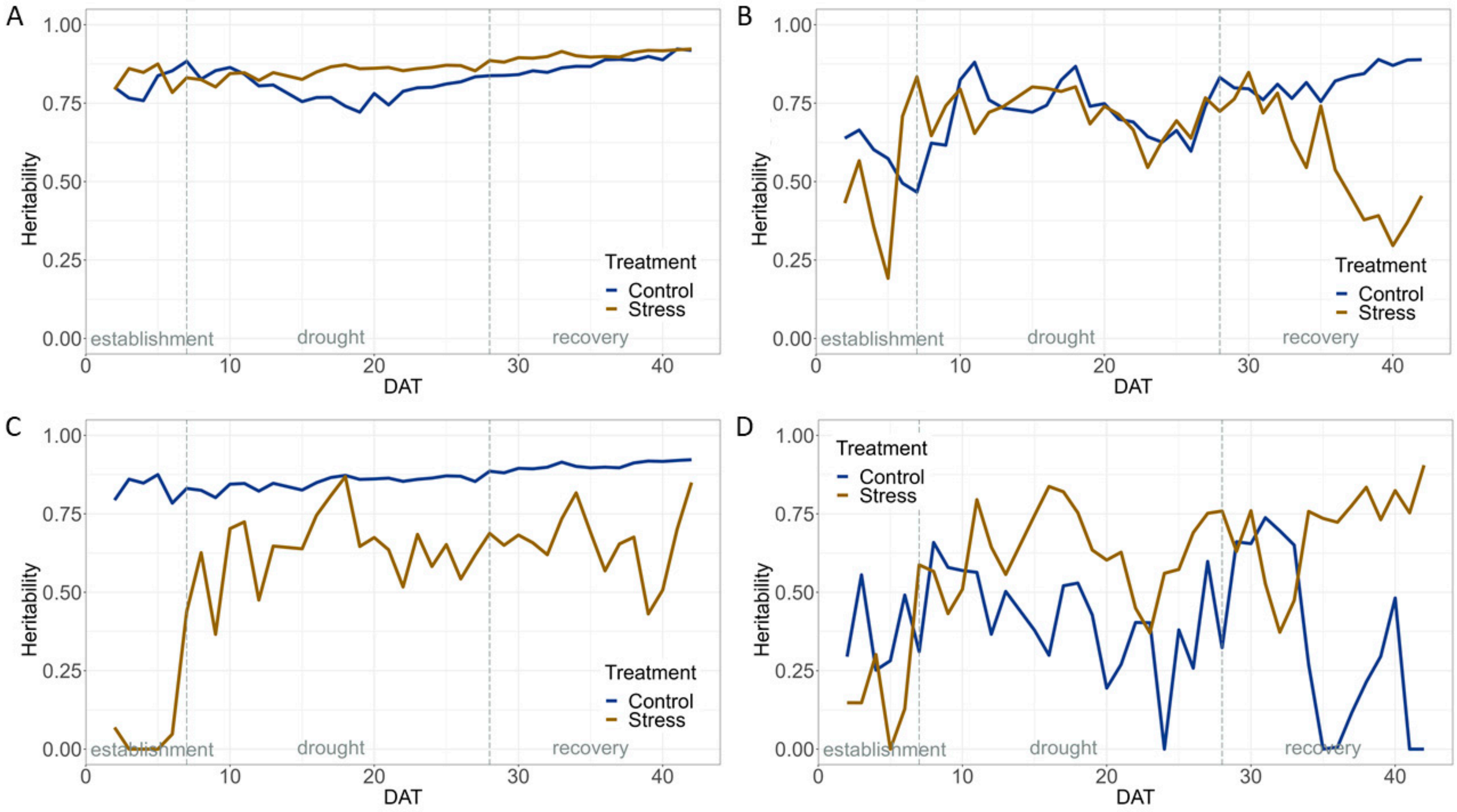

Figure S3: Heritability of Chlorophyll Fluorescence traits

. A) ΦPSIIh; B) ΦPSIIl C) ΦPSIIr;  ΦPSIIl = operating efficiency under low light conditions; ΦPSIIh = operating efficiency under low light conditions; ΦPSIIr  = (ΦPSIIl / ΦPSIIh). 1 day before drought= DAT 6, 7 days of drought=DAT13, 13 days of drought = DAT20, 19 days of drought = DAT 27, 6 days of recovery = DAT34.

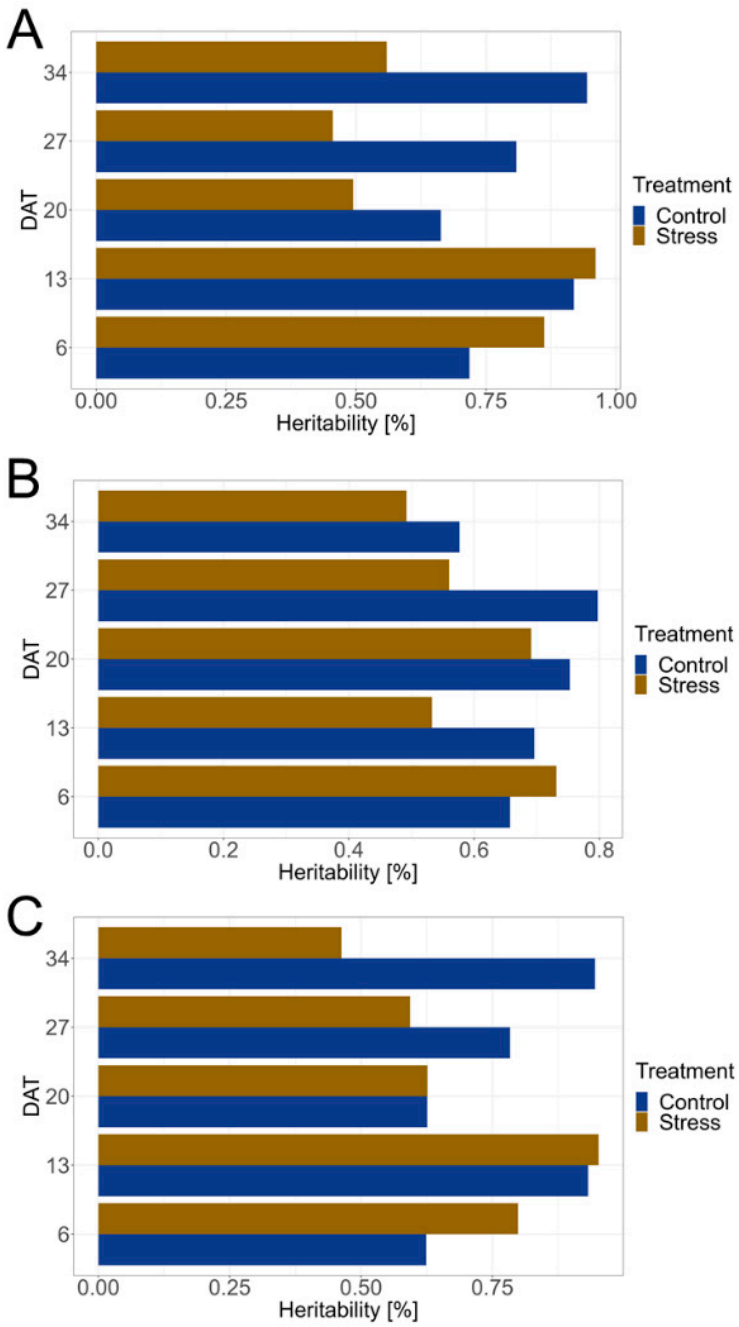

Figure S4: Correlation of EB from DAT 42 and fresh weight from DAT 43

. Based on BLUEs across both experiments of all 60 genotypes. *p* indicates the level of significance and *r* the coefficient of correlation.

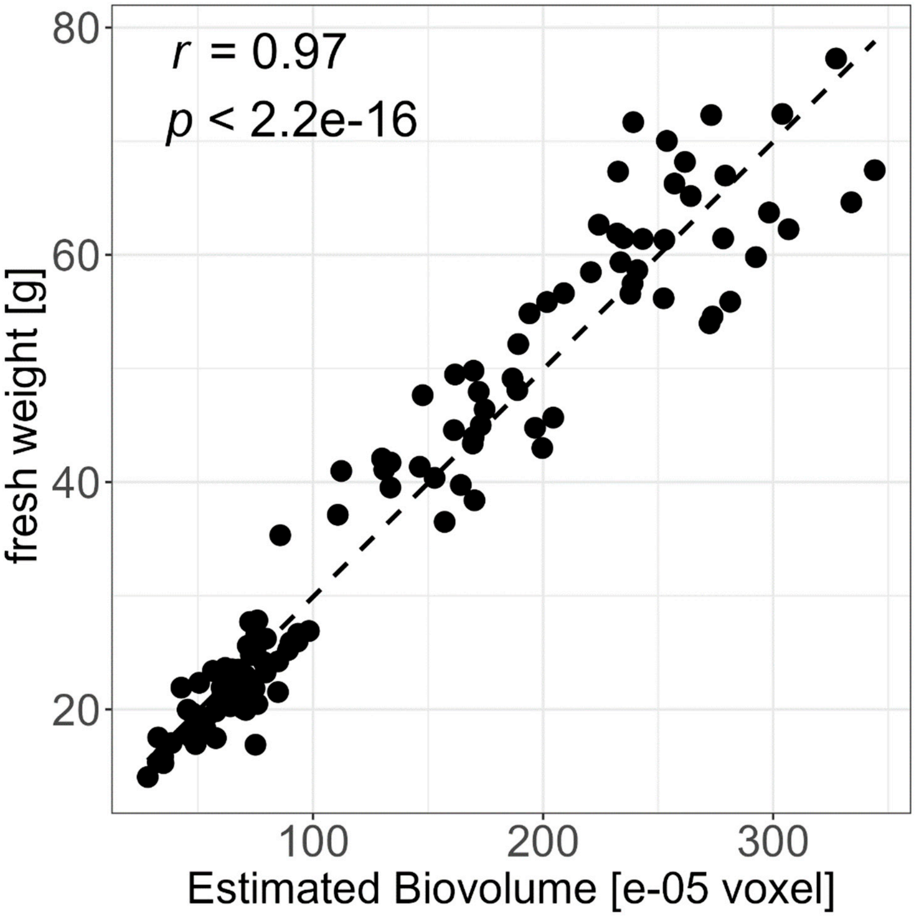

Figure S5: Relative growth rate of drought stress treatment.

Based on the mean value of the BLUEs across both experiments for all 60 genotypes. The second axis with the dotted line shows the plant available water (PAW). RGR [voxel /DAT] = relative growth rate = $(\ln(EB_i)-\ln(EB_{i-1}))/(DAT_i-DAT_{i-1})$

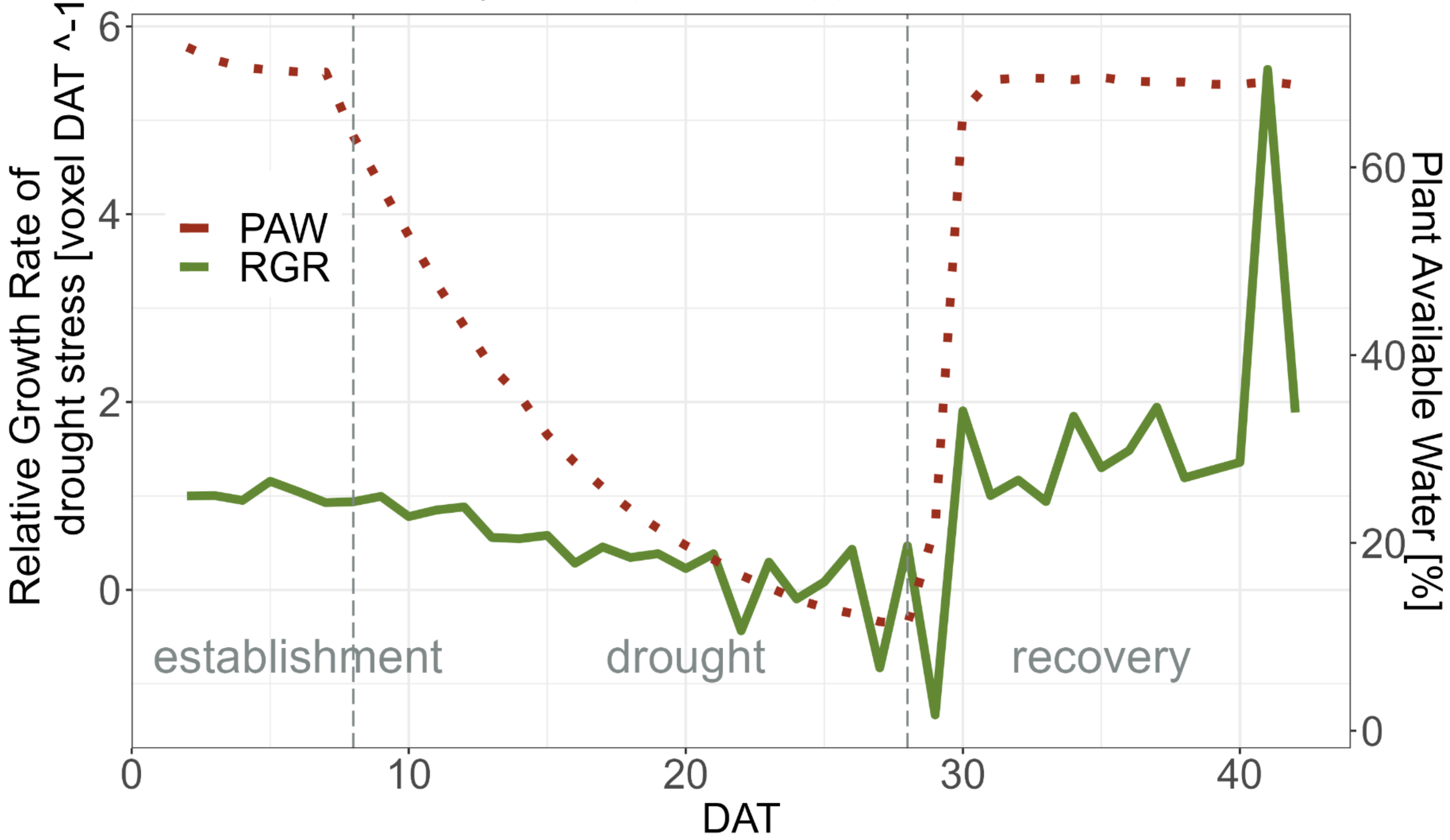

## Figure S6: Coefficient of variation of image-derived traits

Based on BLUEs across both experiments and all 60 genotypes. DAT on x axis. Establishment phase = DAT 2-7; drought stress = DAT8-28; recovery = DAT 29-42. A) EB; B) PH; C) MCV; D) r2g; E) y2g.

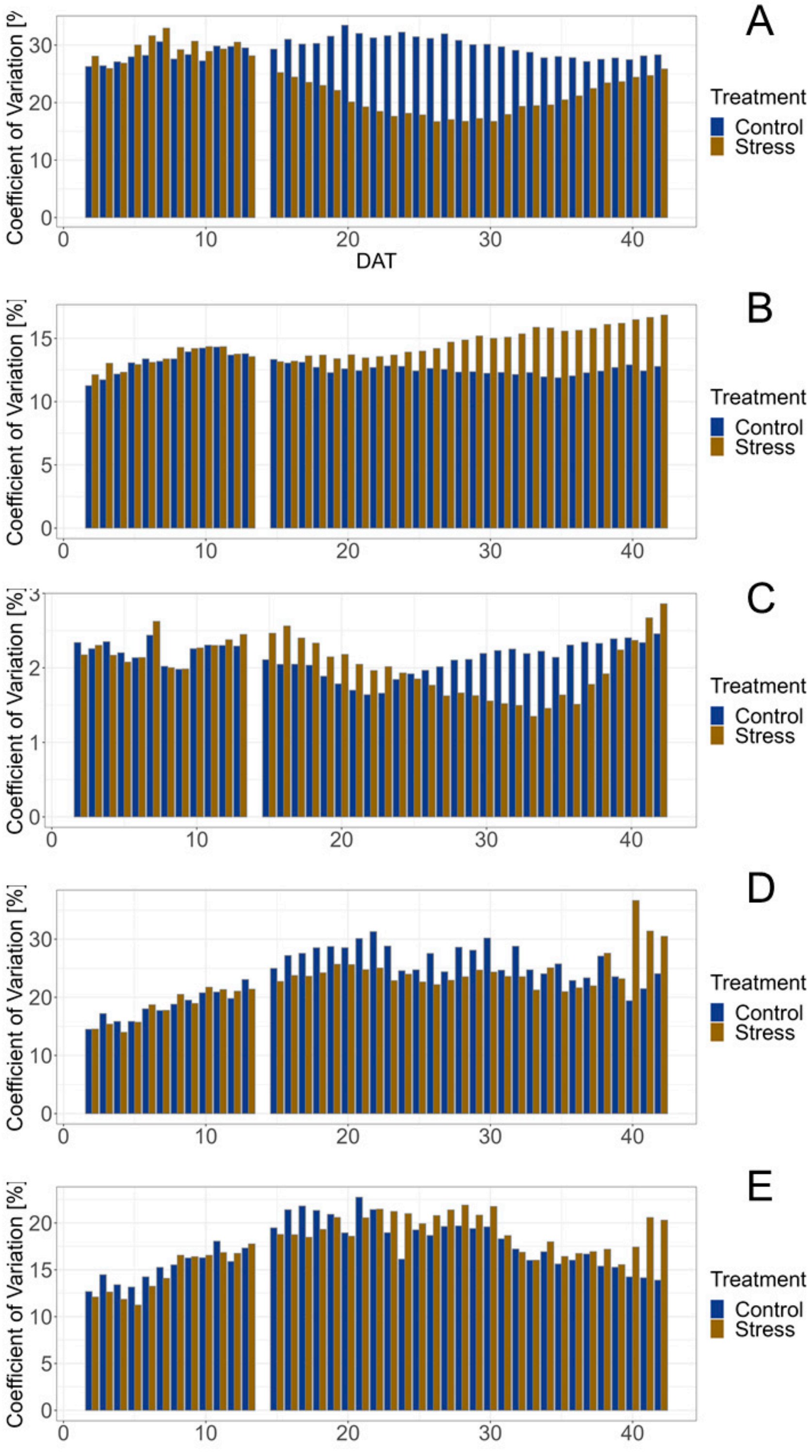

## Figure S7: Chlorophyll Fluorescence traits for drought phases

Based on BLUEs across for both experiments and on all 60 genotypes. The shape around the boxplot is a violin plot and describes the continuous distribution of the data at different values.

A) ΦPSIIl. B) ΦPSIIr; 1 day before drought= DAT 6, 7 days of drought=DAT13, 13 days of drought = DAT20, 19 days of drought = DAT 27, 6 days of recovery = DAT34. ΦPSII means operating efficiency of photosystem II under high (h) and low (l) light condition; (r) means the ΦPSIIl / ΦPSIIh ratio.

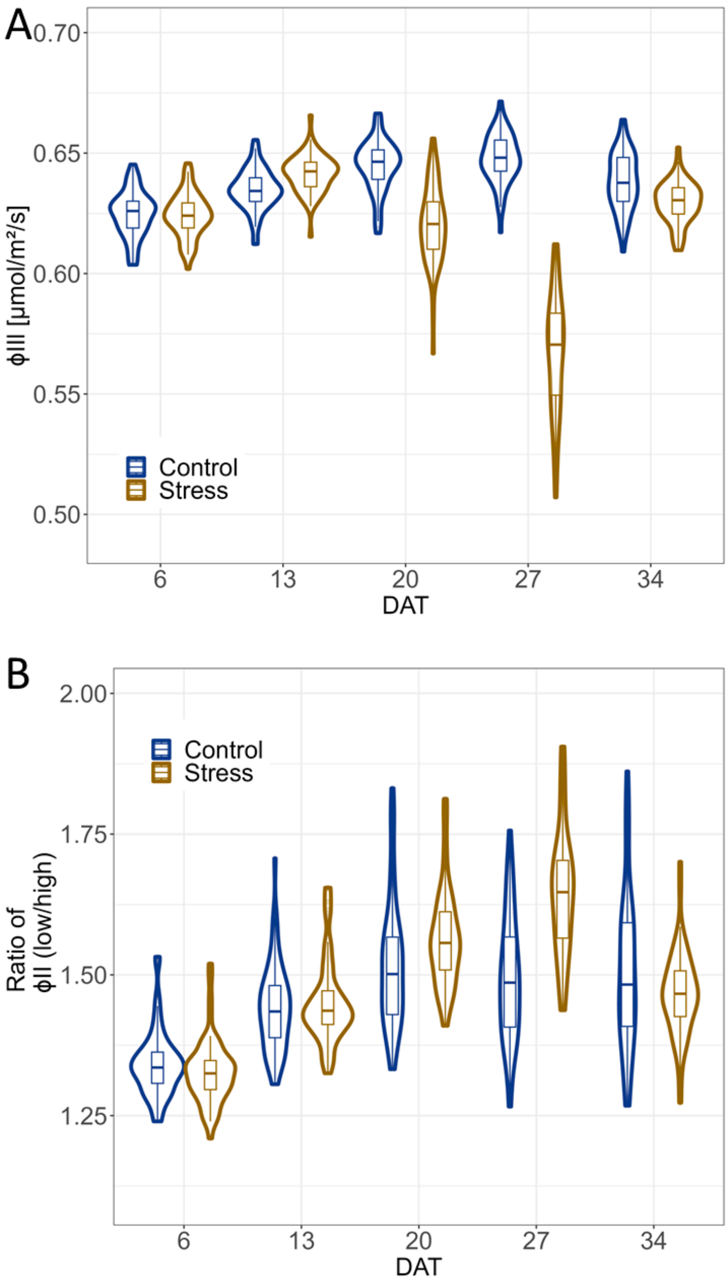

Figure S8: Correlation among chlorophyll fluorescence traits

Based on BLUEs across both experiments and all 60 genotypes. *p* indicates the level of significance and *r* the coefficient of correlation. Between A.) ΦPSIIr and ΦPSIIh; B.) ΦPSIIl and ΦPSIIh and C.) ΦPSIIl and ΦPSIIr. ΦPSII means operating efficiency of photosystem II under high (h) and low (l) light condition; (r) means the ΦPSIIl / ΦPSIIh ratio.

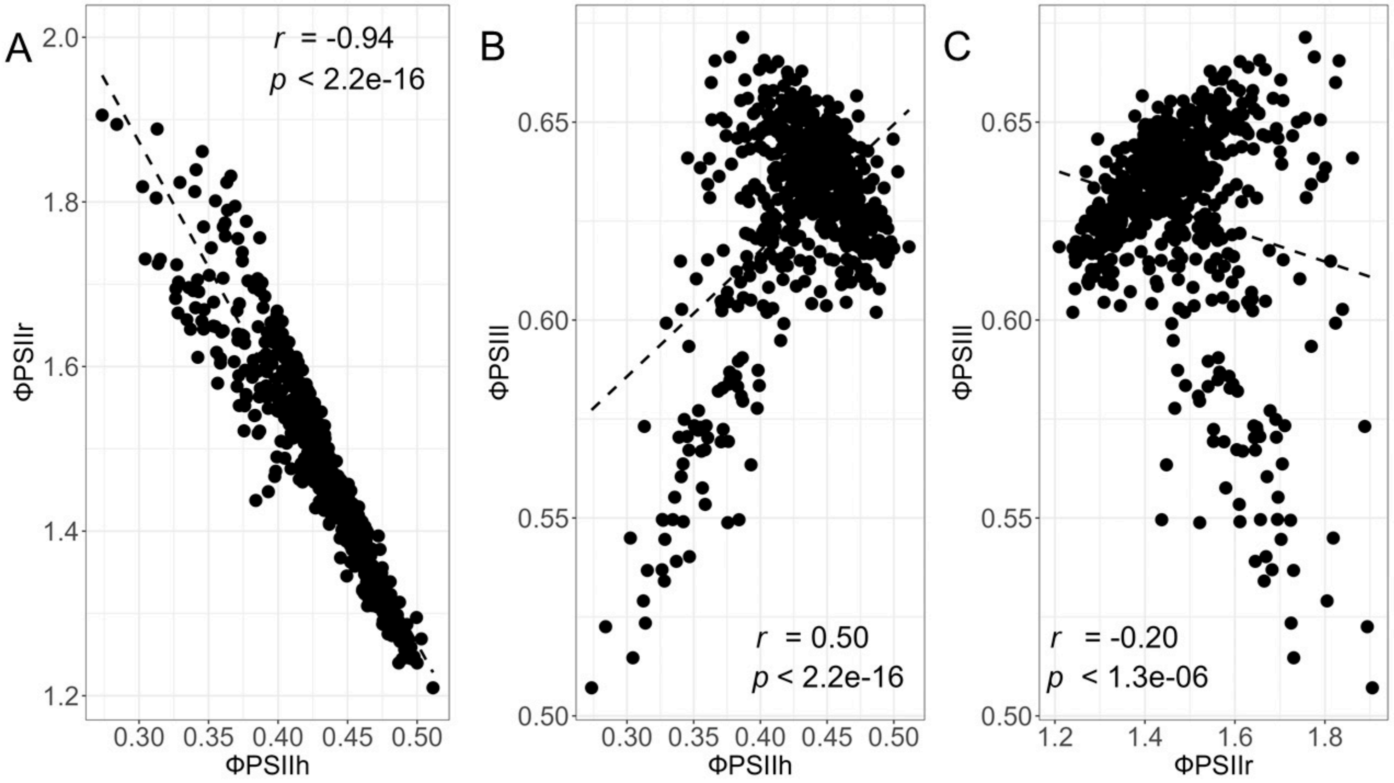

## Figure S9: Correlation of ΦPSIIh and image-derived traits for DAT 27.

Based on both treatments and BLUEs across both experiments of all 60 genotypes. All correlations are significant with $p < 0.001$. r = coefficient of correlation; $R^2$ = coefficient of determination; y=linear regression.

A) EB; B) PH; C) MCV; D) r2g; E) y2g.

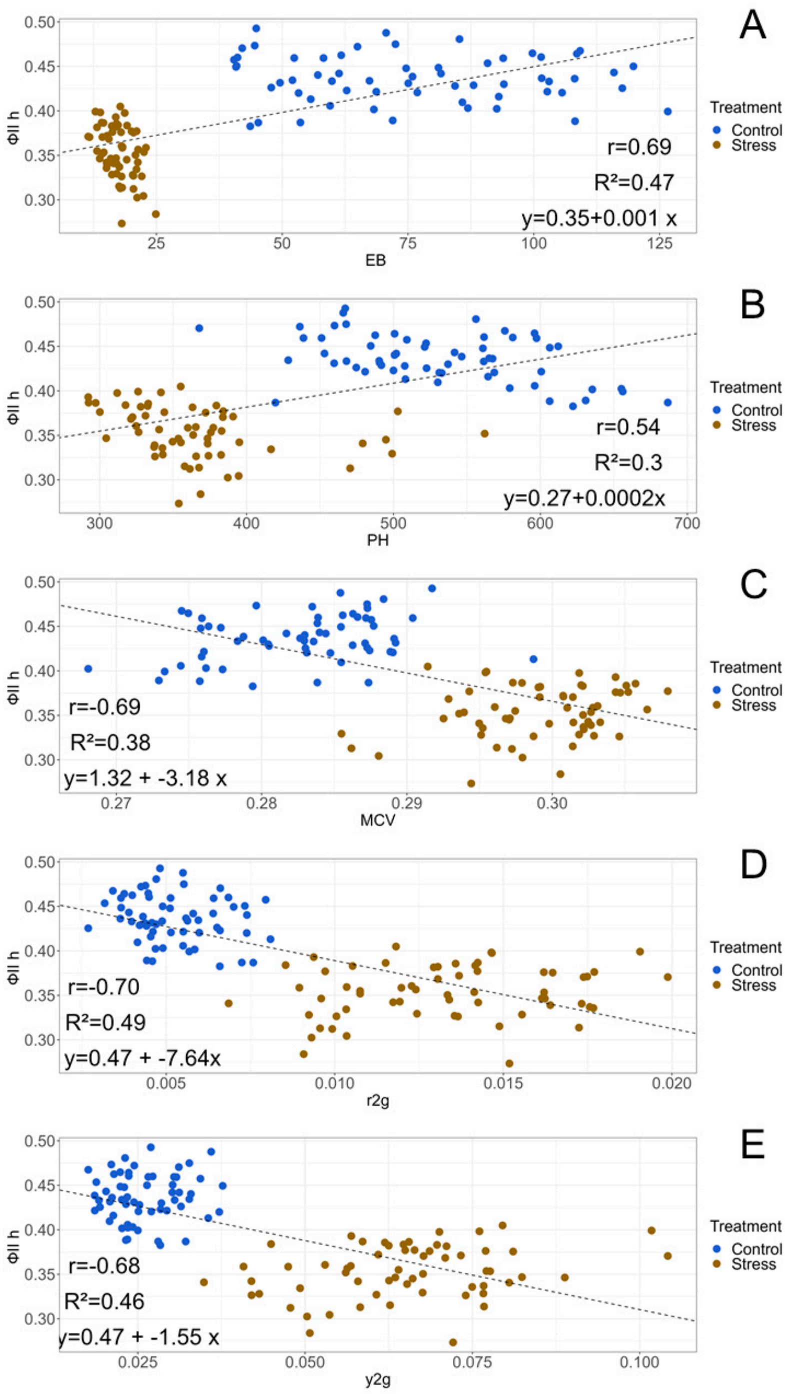

## Figure S10: Correlation matrix of Chlorophyll Fluorescence and image-derived traits

A) DAT6; B) DAT13; C) DAT20; D) DAT27; E) DAT34.

Based on both treatments and BLUEs across both experiments and all 60 genotypes.

DAT 6 = 1 day before drought, DAT 13 = 7 days of drought, DAT 20 = 13 days of drought, DAT 27 = 19 days of drought, DAT 34 = 6 days of recovery. ϕPSIIh = operating efficiency of photosystem II under high light conditions.

Boxes with a red color indicate a strong positive correlation and with a purple correlation a strong negative correlation. Displayed are coefficients of correlation *r* which are significant with maximal *p-value* $< 0.05$.

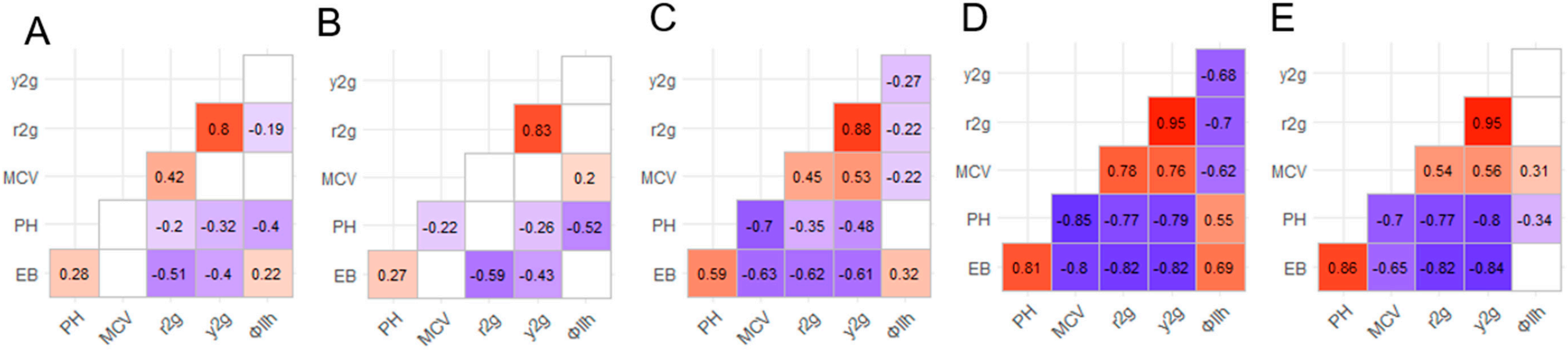

Figure S11: PCA for desi and kabuli for DT.

Based on BLUEs across both experiments

A) Control. B) Stress. DT= drought tolerance DAT8-28.

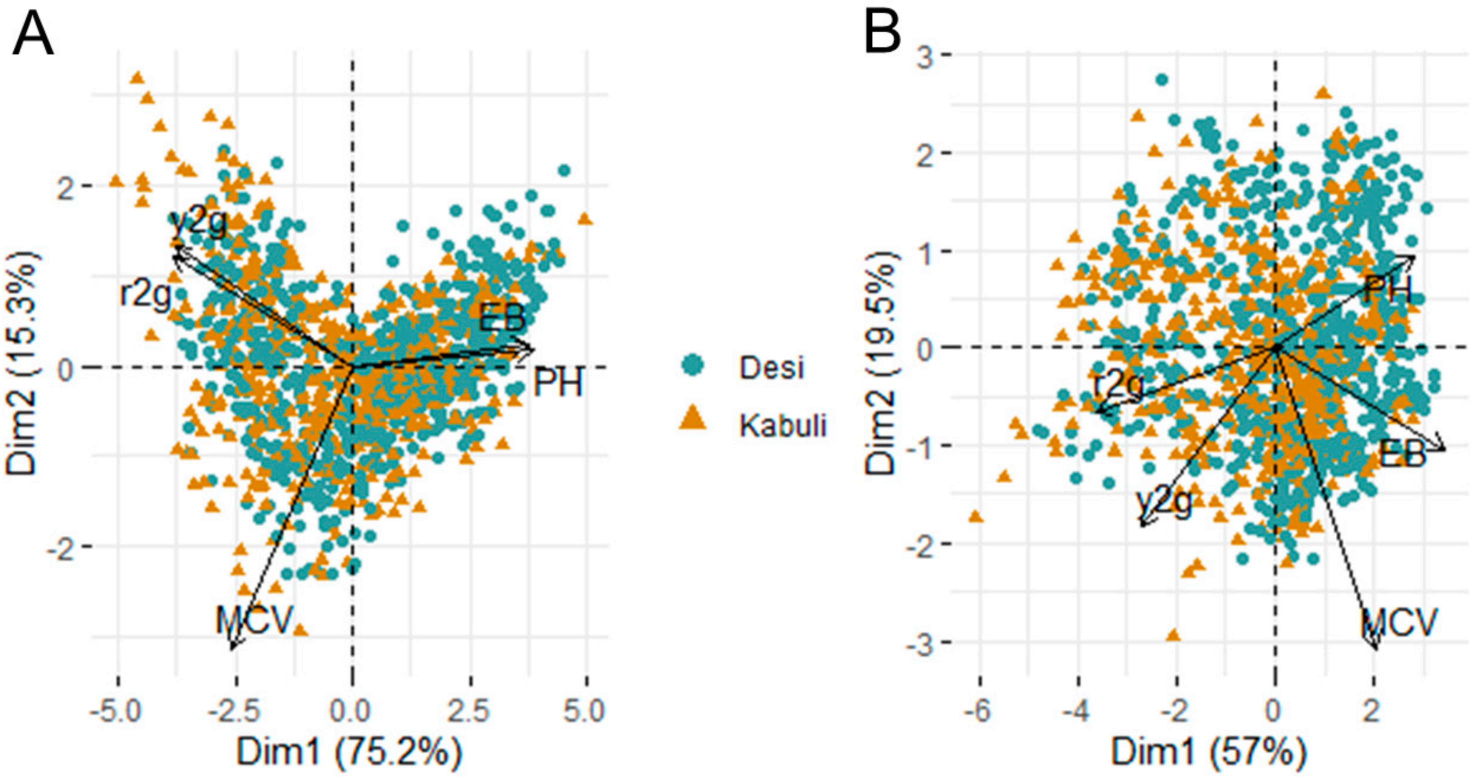

Figure S 12: PCA for DT for biological status

A) Control; B) Stress. DT= drought tolerance DAT8-28.

Based on BLUEs across both experiments; grouped for the biological status.

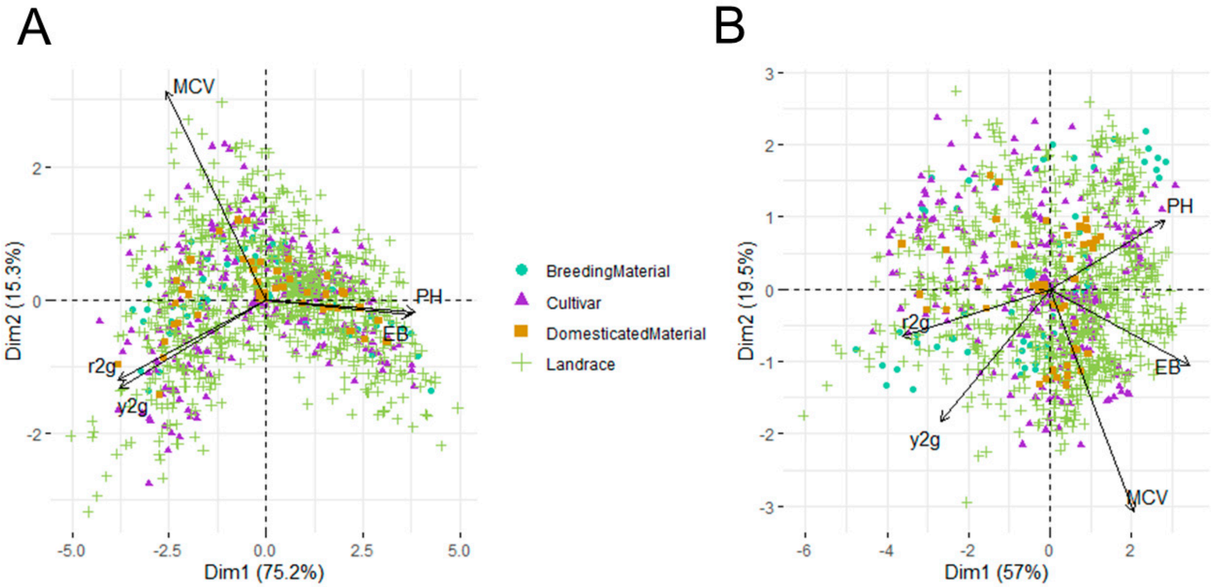

Figure S13: Loss of drought stress compared to control treatment of *desi* and *kabuli*

A) EB; B) PH; C) MCV; D) r2g; E) y2g. Based on BLUEs across both experiments. The shadows describe the 95 % confidence interval; as long as the shadows do not overlap, the significance level of $\alpha$=0.05 was reached and therefore a significant difference exists. Loss [%] = (1-(stress/control))*100.

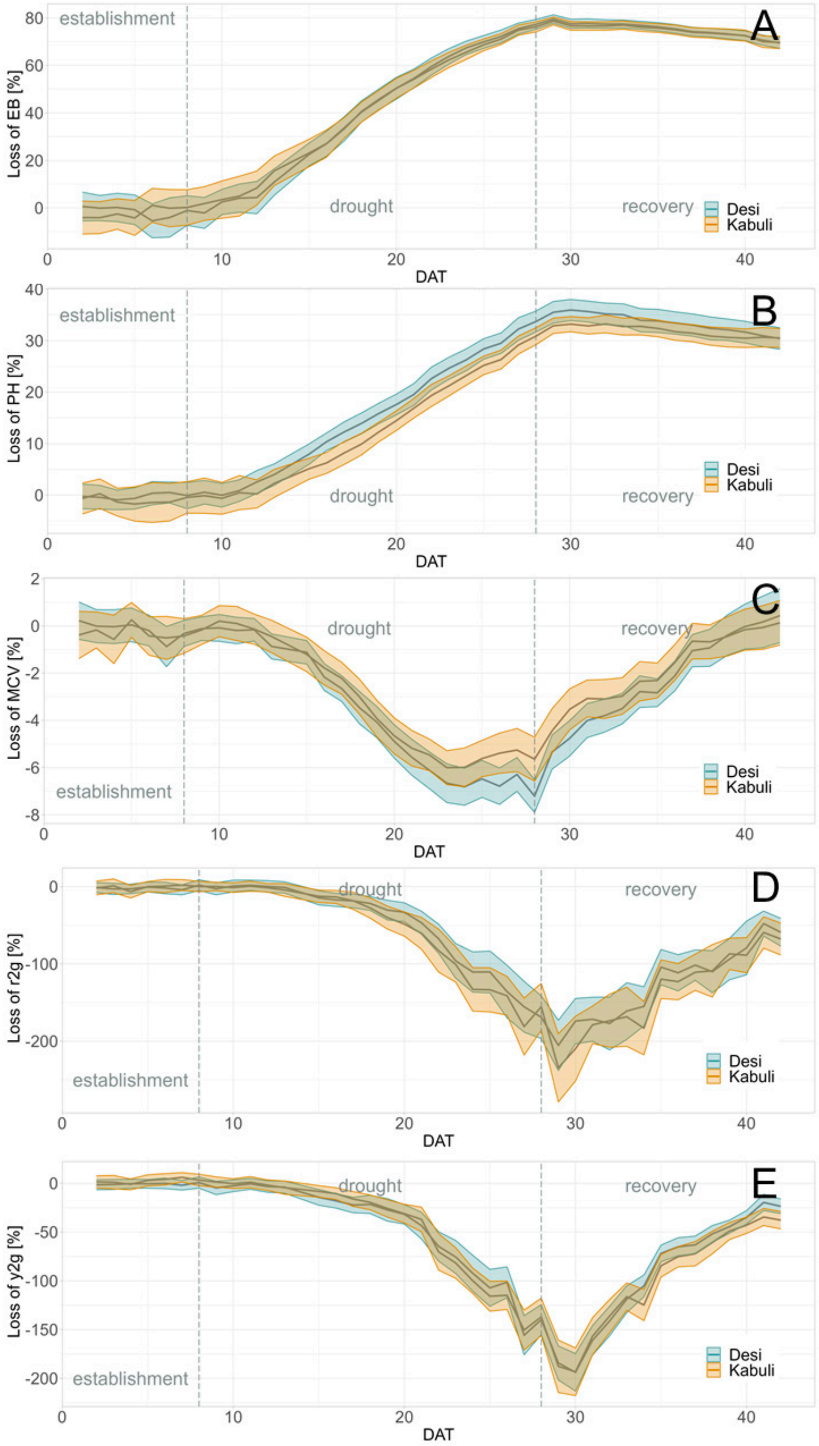

## Figure S 14: Loss of drought stress compared to control treatment of biological status

A) EB; B) PH; C) MCV; D) r2g; E) y2g. Based on BLUEs across and all genotypes. The shadows describe the 95 % confidence interval; as long as the shadows do not overlap, the significance level of $\alpha$=0.05 was reached and therefore a significant difference exists. Loss [%] = (1-(stress/control))*100.

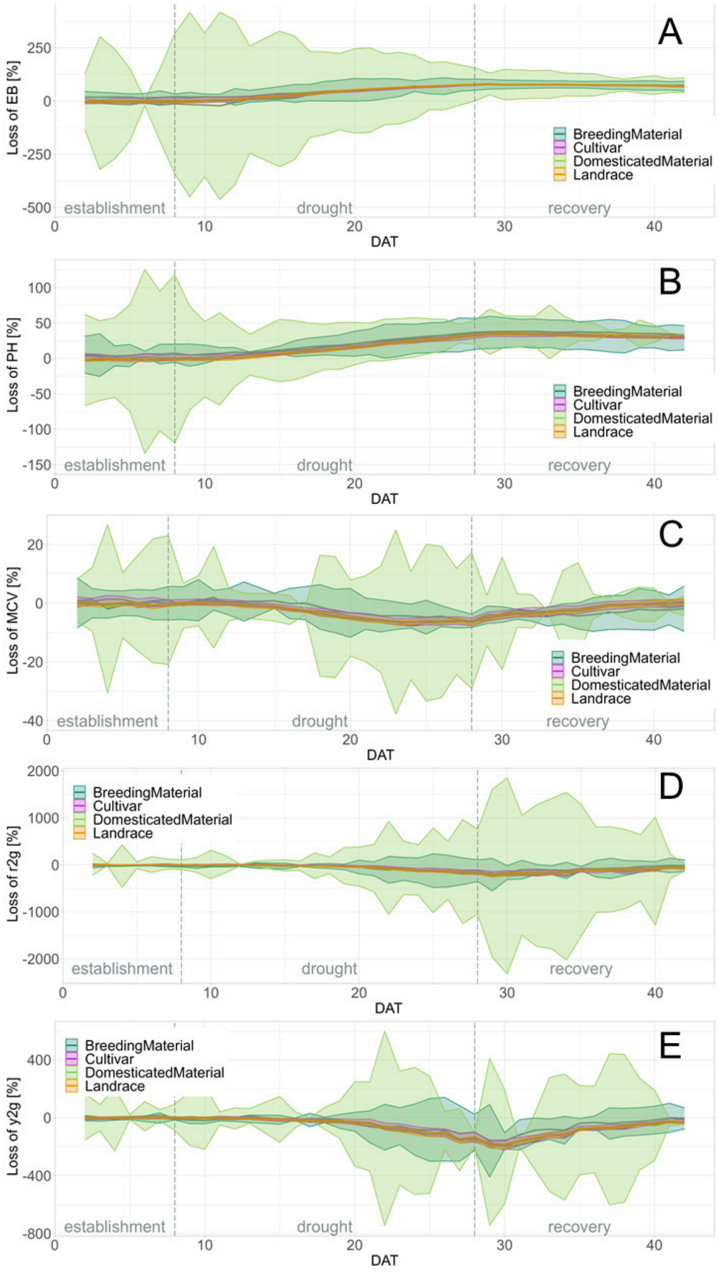

Figure S 15: Deviation of the individual genotypes with their affiliation to the biological status, from the mean value of EB of all 60 genotypes.
Based on BLUEs across both experiments and all 60 genotypes. A) DAT 28; B) DAT42.

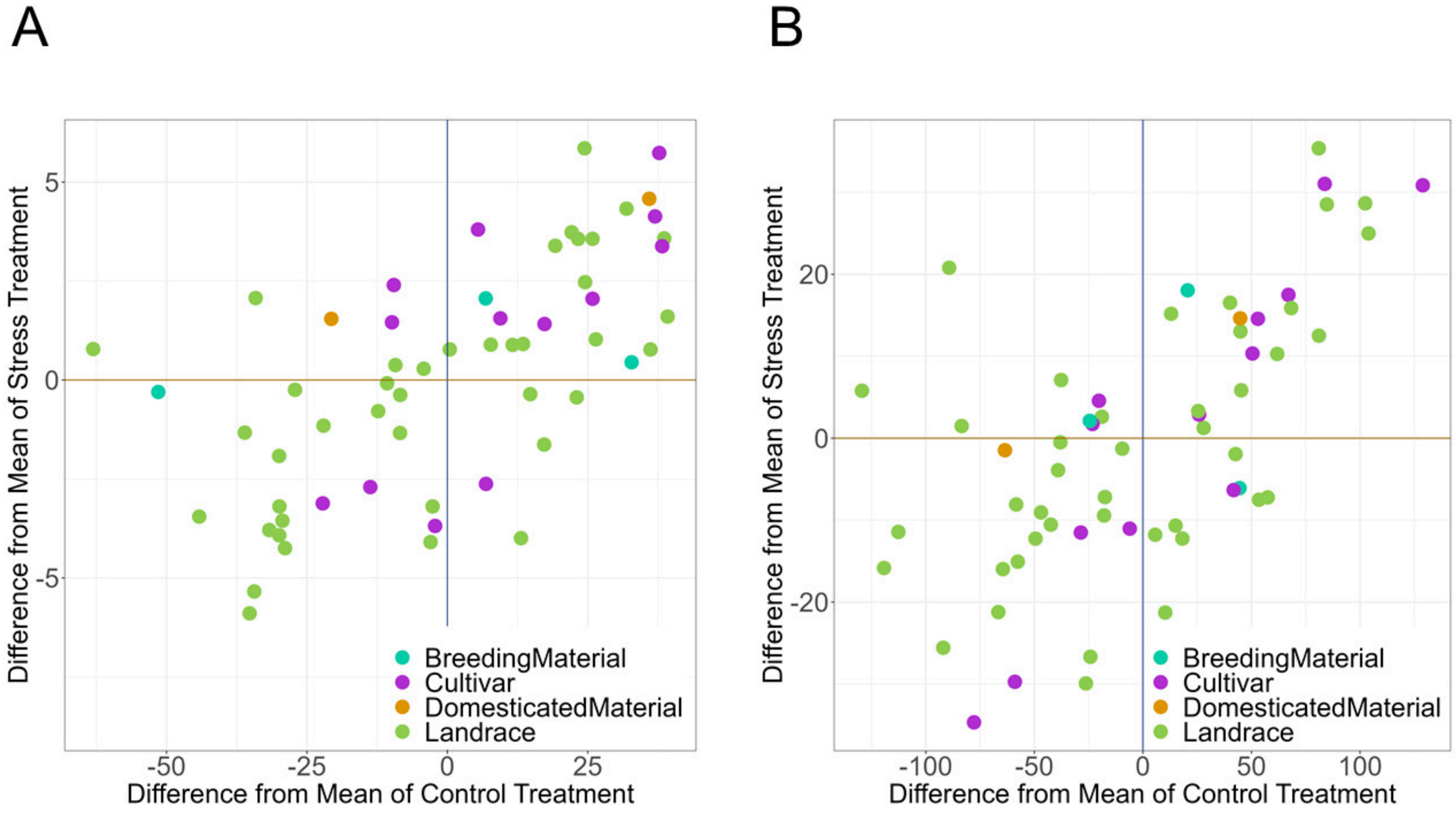

## Figure S 16: WUE of *desi* and *kabuli* during drought phases

Based on BLUEs across both experiments for EB. The shape around the boxplot is a violin plot and describes the continuous distribution of the data at different values. A) *desi* and *kabuli* for DA; B) *desi* and *kabuli* for DR. DA= drought adaptability DAT 8-42 and DR= drought recovery DAT 29-42.

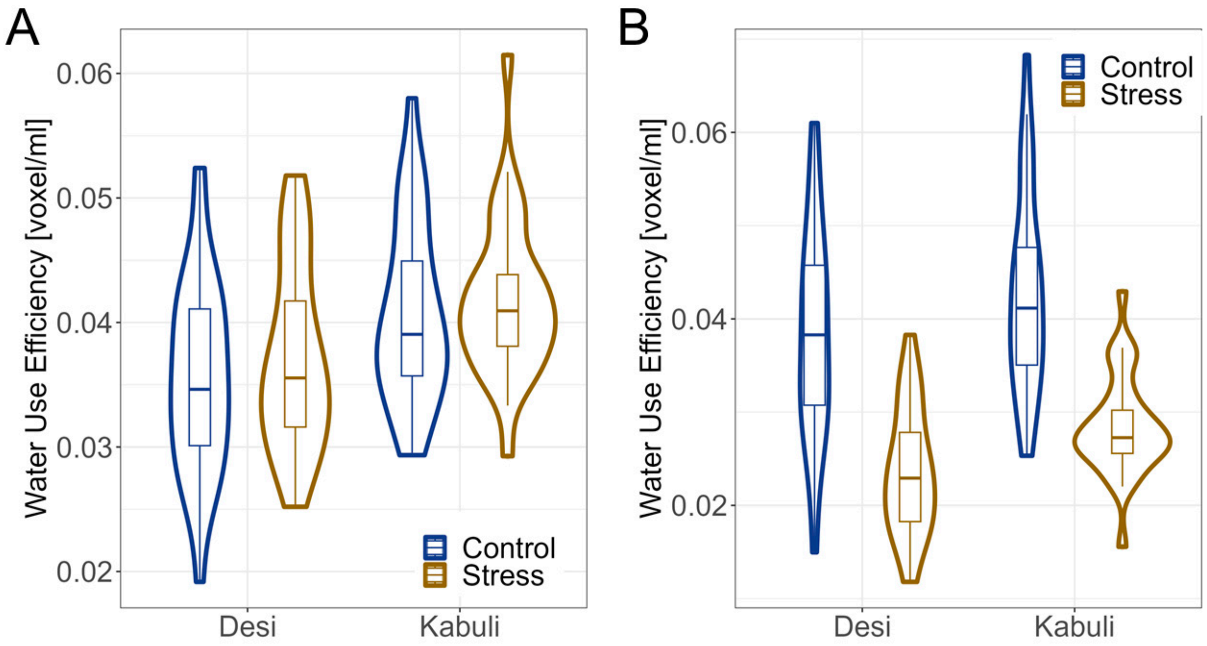

Figure S 17: Deviation of the individual genotypes with their affiliation to *desi* and *kabuli*, from the mean value of all 60 genotypes.

Based on BLUEs across both experiments and all 60 genotypes

A.) MCV DAT 28; B.) MCV DAT 42; C) y2g DAT 28, D) y2g DAT42; E) r2g DAT 28; F) 2g DAT42. DAT 28 = Day after transferring with the maximum period of drought stress; DAT 42 = Day after transferring with the longest period of recovery.

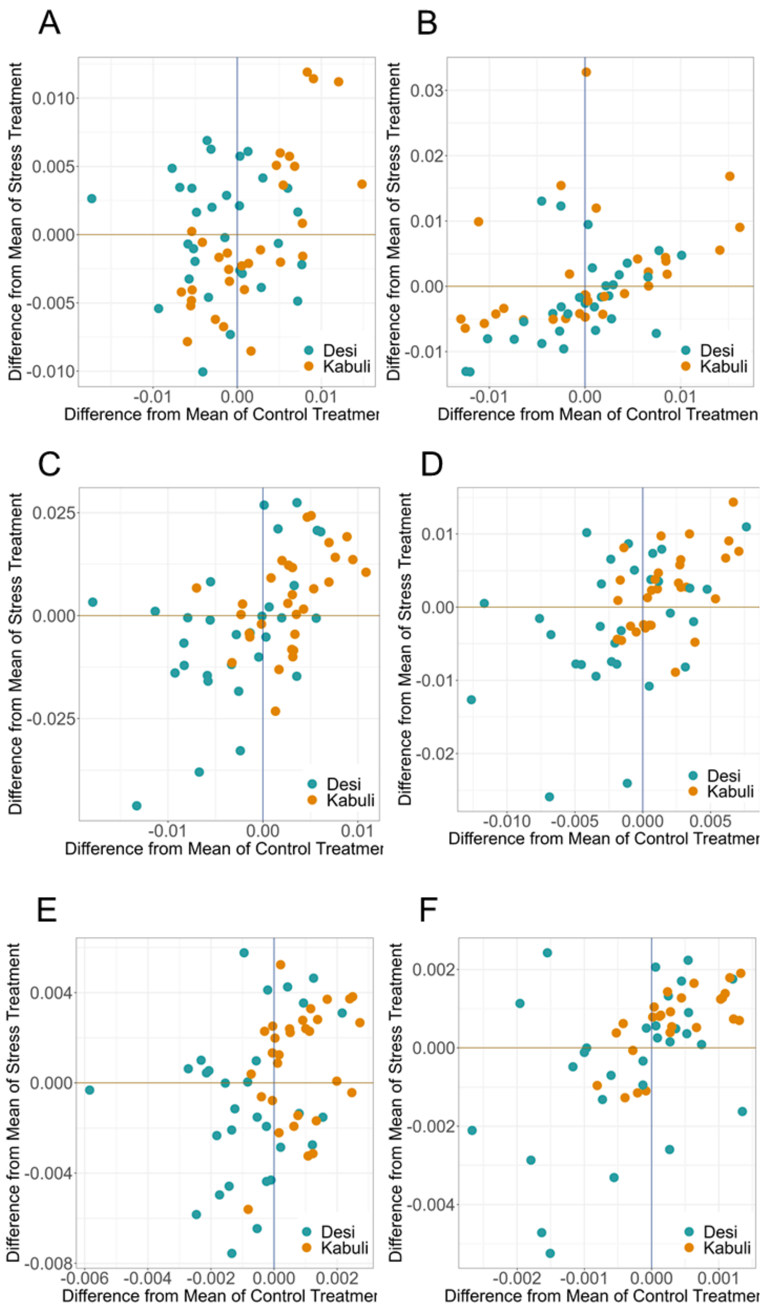

Figure S 18: Deviation of the individual genotypes from the mean value of ϕ PS IIh of all 60 genotypes

Based on BLUEs across both experiments and all 60 genotypes. DAT 27 =19 DATs of drought stress and DAT 34 = 6 DATs of recovery.

A.) *desi* and *kabuli* on DAT 27; B) *desi* and *kabuli* on DAT 34; C) biological status on DAT 27; D) biological status on DAT 34.

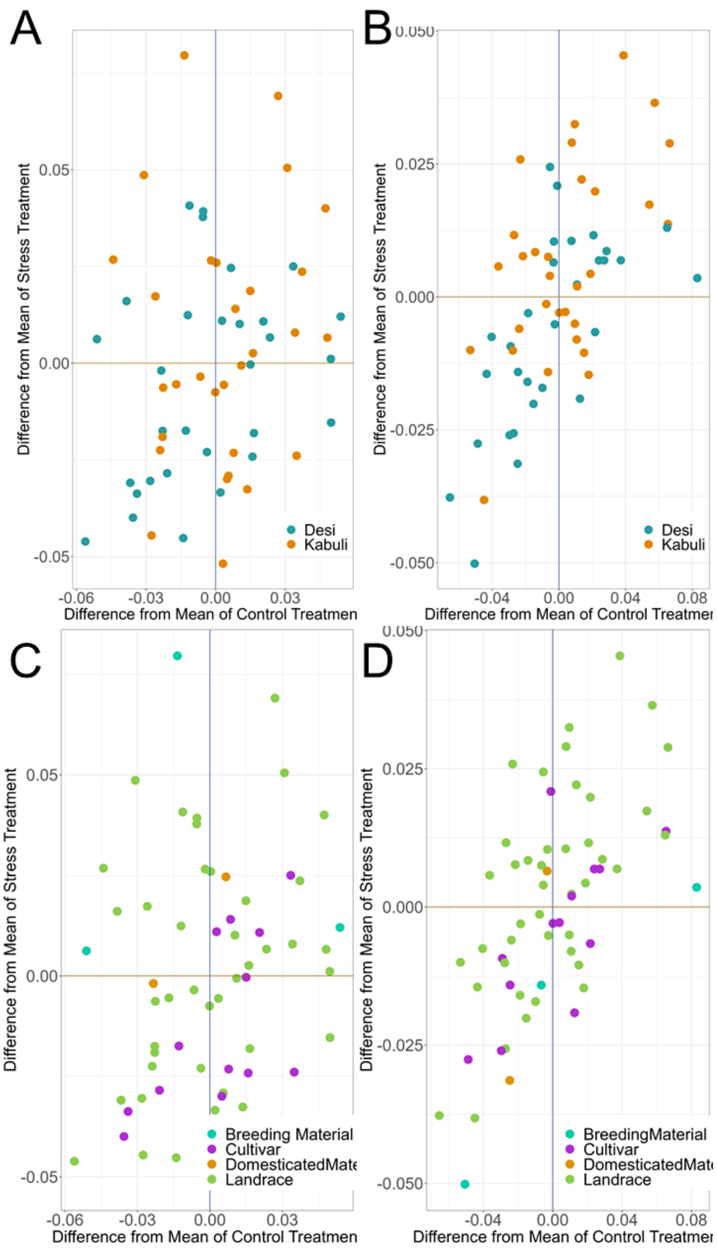

Supplement: Supplementary file 1 [file plants-12-02866-s001.zip › plants-2456699_ supplementary Figure S.pdf]
