# Supplementary material for: Engaging Precision Phenotyping to Scrutinize Vegetative Drought Tolerance and Recovery in Chickpea Plant Genetic Resources
Source: Plants (Basel). 2023 Aug 4;12(15):2866. doi: 10.3390/plants12152866 (PMC10421427; doi:10.3390/plants12152866)
Supplement: Supplementary file 1 [file plants-12-02866-s001.zip › plants-2456699_ supplementary Table S.pdf]

Tables S

Contents

Tables S 1

Table S1: PAW for experiment 1 and 2 and both experiments. 2

Table S2: Descriptive statistics of image-derived traits for all genotypes and *desi* and *kabuli*. 4

Table S3: Descriptive statistics of fresh and dry weight. 9

Table S4: Descriptive statistics of RGR. 10

Table S5: Descriptive statistics of WUE for drought phases and chickpea types.................12

Table S6: Tukey`s test of WUE in different drought phases and for desi and kabuli under drought phase. 13

Table S7: Descriptive statistics of Chlorophyll Fluorescence traits. 15

Table S8: Tukey's test for chlorophyll fluorescence traits 18

Table S9: Correlations of EB with further image-derived traits during the period of DA, DR and DT. 20

Table S10: Correlation between ΦPSIIh and image-derived traits. 21

Table S11: Superior genotypes for EB and WUE as measured by the difference from the mean of the panel under drought stress compared to the control treatment. 22

Table S12: Description of Plant Material. 26

Table S1: PAW for experiment 1 and 2 and both experiments.

Means among all 60 genotypes. PAW = plant available water

	experiment 1		experiment 2		both experiments	
DAT	control	stress	control	stress	control	stress
1	78.3	77.6	68.8	70.8	73.5	74.2
2	75.7	75.2	70.5	70.4	73.1	72.8
3	73.3	72.8	70.0	70.0	71.7	71.4
4	71.6	71.4	69.9	69.9	70.7	70.6
5	70.7	70.8	69.8	69.9	70.2	70.3
6	70.4	70.5	69.8	69.8	70.1	70.2
7	70.4	70.4	69.9	69.9	70.1	70.1
8	70.7	61.3	69.7	65.3	70.2	63.3
9	70.6	54.8	77.5	61.2	74.0	58.0
10	70.6	48.4	78.2	57.0	74.4	52.7
11	70.6	42.7	78.3	52.6	74.5	47.6
12	70.7	37.5	78.3	48.7	74.5	43.1
13	70.7	32.7	78.3	44.7	74.5	38.7
14	70.5	29.7	78.3	41.7	74.4	35.7
15	70.8	25.6	78.0	37.4	74.4	31.5
16	70.6	23.1	78.1	34.0	74.3	28.6
17	70.7	20.7	78.1	31.1	74.4	25.9
18	70.7	18.6	78.0	28.5	74.4	23.5
19	70.6	17.0	77.9	26.1	74.3	21.5
20	70.5	15.6	77.9	23.8	74.2	19.7
21	70.5	14.4	78.0	22.1	74.3	18.2
22	70.4	13.5	77.4	19.6	73.9	16.5
23	70.4	12.7	77.3	17.8	73.9	15.3
24	70.5	11.9	77.2	16.1	73.8	14.0
25	70.2	11.4	77.3	14.8	73.8	13.1
26	70.1	11.1	77.5	13.9	73.8	12.5
27	70.1	10.8	76.4	12.4	73.2	11.6
28	70.0	10.7	77.5	12.0	73.8	11.3
29	69.1	10.5	68.6	33.1	68.9	21.8
30	69.0	67.5	67.8	64.3	68.4	65.9
31	69.3	69.9	68.0	68.7	68.6	69.3
32	69.1	69.9	67.7	69.1	68.4	69.5
33	68.8	69.9	67.6	69.0	68.2	69.5
34	68.8	69.8	67.1	68.8	68.0	69.3
35	69.1	69.9	68.3	69.2	68.7	69.6
36	69.0	69.9	66.9	68.5	67.9	69.2

37	68.6	69.7	66.8	68.4	67.7	69.1
38	68.9	69.8	66.8	68.3	67.9	69.1
39	68.7	69.8	66.5	67.9	67.6	68.8
40	68.6	69.7	67.0	68.2	67.7	68.8
41	68.4	69.6	67.5	68.7	68.0	69.1
42	68.9	69.7	66.3	67.8	67.6	68.8

Table S2: Descriptive statistics of image-derived traits for all genotypes and *desi* and *kabuli*.

Based on BLUEs across both experiments and all 60 genotypes. q1= Quantile 25%; q3= Quantile 75%; sd=standard deviation. Loss [%] = (1-(stress/control))*100

trait	DAT	chickpea	treatment	min	q1	median	mean	q3	max	sd
EB	7	all	control	1.83	3.5	4.23	**4.42**	5.32	7.71	1.35
EB	7	all	stress	1.92	3.27	4.6	**4.45**	5.11	8.58	1.47
EB	7	all	loss	-4.92	6.57	-8.75	**-0.68**	3.95	-11.28	-8.89
EB	7	*desi*	control	1.83	2.89	3.56	**3.5**	4.12	5.32	0.83
EB	7	*desi*	stress	1.92	2.98	3.42	**3.61**	4.39	5.86	1.02
EB	7	*desi*	loss	-4.92	-3.11	3.93	**-3.14**	-6.55	-10.15	-22.89
EB	7	*kabuli*	control	3.53	4.5	5.28	**5.33**	5.94	7.71	1.14
EB	7	*kabuli*	stress	2.82	4.67	4.99	**5.29**	6.13	8.58	1.37
EB	7	*kabuli*	loss	20.11	-3.78	5.49	**0.75**	-3.20	-11.28	-20.18
EB	28	all	control	45.94	62.06	85.99	**85.15**	107.23	148.21	26.24
EB	28	all	stress	12.69	16.48	18.13	**18.55**	21.19	27.2	3.11
EB	28	all	loss	72.38	73.45	78.92	**78.21**	80.24	81.65	88.15
EB	28	*desi*	control	45.94	52.57	66.89	**70.53**	85.42	115.11	20.46
EB	28	*desi*	stress	12.69	14.97	16.99	**17.07**	18.72	22.23	2.7
EB	28	*desi*	loss	72.38	71.52	74.60	**75.80**	78.08	80.69	86.80
EB	28	*kabuli*	control	59.3	85.36	96.67	**99.78**	116.41	148.21	23.24
EB	28	*kabuli*	stress	16.07	17.77	19.52	**20.02**	22.28	27.2	2.79
EB	28	*kabuli*	loss	72.90	79.18	79.81	**79.94**	80.86	81.65	87.99
EB	42	all	control	85.76	169.67	214.89	**214.7**	258.24	344.13	60.78
EB	42	all	stress	28.17	50.92	64.45	**63.56**	74.69	98.23	16.43
EB	42	all	loss	67.15	69.99	70.01	**70.40**	71.08	71.46	72.97
EB	42	*desi*	control	85.76	146.71	169.81	**182.1**	201.18	333.96	57.15
EB	42	*desi*	stress	28.17	46.3	52.13	**56.42**	68.61	98.23	17.38
EB	42	*desi*	loss	67.15	68.44	69.30	**69.02**	65.90	70.59	69.59

EB	42	*kabuli*	control	152.84	221.61	246.67	**247.3**	273.52	344.13	45.27
EB	42	*kabuli*	stress	42.76	61.6	72.13	**70.69**	75.81	93.48	11.9
EB	42	*kabuli*	loss	72.02	72.20	70.76	**71.42**	72.28	72.84	73.71
MCV	7	all	control	0.266	0.275	0.279	**0.279**	0.283	0.295	0.007
MCV	7	all	stress	0.263	0.277	0.28	**0.281**	0.286	0.298	0.007
MCV	7	all	loss	1.13	-0.73	-0.36	**-0.72**	-1.06	-1.02	0.00
MCV	7	*desi*	control	0.267	0.272	0.278	**0.279**	0.284	0.295	0.008
MCV	7	*desi*	stress	0.264	0.276	0.282	**0.282**	0.288	0.295	0.008
MCV	7	*desi*	loss	1.12	-1.47	-1.44	**-1.08**	-1.41	0.00	0.00
MCV	7	*kabuli*	control	0.266	0.276	0.28	**0.28**	0.283	0.292	0.006
MCV	7	*kabuli*	stress	0.263	0.277	0.28	**0.28**	0.283	0.298	0.007
MCV	7	*kabuli*	loss	1.13	-0.36	0.00	**0.00**	0.00	-2.05	-16.67
MCV	28	all	control	0.265	0.275	0.28	**0.28**	0.284	0.297	0.006
MCV	28	all	stress	0.286	0.294	0.299	**0.298**	0.301	0.308	0.005
MCV	28	all	loss	-7.92	-6.91	-6.79	**-6.43**	-5.99	-3.70	16.67
MCV	28	*desi*	control	0.272	0.278	0.282	**0.281**	0.285	0.297	0.006
MCV	28	*desi*	stress	0.291	0.294	0.297	**0.297**	0.3	0.308	0.004
MCV	28	*desi*	loss	-6.99	-5.76	-5.32	**-5.69**	-5.26	-3.70	33.33
MCV	28	*kabuli*	control	0.265	0.274	0.278	**0.278**	0.282	0.286	0.006
MCV	28	*kabuli*	stress	0.286	0.294	0.299	**0.298**	0.302	0.306	0.006
MCV	28	*kabuli*	loss	-7.92	-7.30	-7.55	**-7.19**	-7.09	-6.99	0.00
MCV	42	all	control	0.261	0.274	0.277	**0.277**	0.281	0.29	0.007
MCV	42	all	stress	0.244	0.273	0.278	**0.276**	0.281	0.29	0.008
MCV	42	all	loss	6.51	0.36	-0.36	**0.36**	0.00	0.00	-14.29
MCV	42	*desi*	control	0.267	0.275	0.277	**0.278**	0.281	0.29	0.006
MCV	42	*desi*	stress	0.263	0.275	0.279	**0.278**	0.283	0.29	0.007
MCV	42	*desi*	loss	1.50	0.00	-0.72	**0.00**	-0.71	0.00	-16.67
MCV	42	*kabuli*	control	0.261	0.271	0.277	**0.277**	0.281	0.29	0.008
MCV	42	*kabuli*	stress	0.244	0.272	0.278	**0.275**	0.281	0.283	0.009
MCV	42	*kabuli*	loss	6.51	-0.37	-0.36	**0.72**	0.00	2.41	-12.50

PH	7	all	control	182.97	213.93	233.81	**237.02**	256.01	317.77	31.26
PH	7	all	stress	176.91	215.59	235.37	**237.26**	252.12	334.52	31.72
PH	7	all	loss	3.31	-0.78	-0.67	**-0.10**	1.52	-5.27	-1.47
PH	7	*desi*	control	182.97	207.69	228.5	**230.85**	253.92	302.11	29.71
PH	7	*desi*	stress	176.91	210.27	231.5	**229.37**	244.68	286.54	27.06
PH	7	*desi*	loss	3.31	-1.24	-1.31	**0.64**	3.64	5.15	8.92
PH	7	*kabuli*	control	189.22	220.93	240.44	**243.19**	257.98	317.77	32.03
PH	7	*kabuli*	stress	194.92	223.94	240.76	**245.16**	260.48	334.52	34.44
PH	7	*kabuli*	loss	-3.01	-1.36	-0.13	**-0.81**	-0.97	-5.27	-7.52
PH	28	all	control	380.2	493.1	539.62	**544.82**	589.09	697.22	67.18
PH	28	all	stress	291.66	335.68	361.64	**367.9**	379.32	561.46	54.7
PH	28	all	loss	23.29	31.92	32.98	**32.47**	35.61	19.47	18.58
PH	28	*desi*	control	380.2	479.07	515.64	**524.12**	575.27	697.22	67.58
PH	28	*desi*	stress	291.66	328.73	357.27	**361.19**	379.42	561.46	53.73
PH	28	*desi*	loss	23.29	31.38	30.71	**31.09**	34.04	19.47	20.49
PH	28	*kabuli*	control	445.1	513.07	561.15	**565.52**	615.76	671.13	61.06
PH	28	*kabuli*	stress	301.47	337.01	364.52	**374.62**	379.09	505.74	55.74
PH	28	*kabuli*	loss	32.27	34.32	35.04	**33.76**	38.44	24.64	8.71
PH	42	all	control	454.4	638.33	683.08	**695.9**	743.27	908.82	88.93
PH	42	all	stress	335.11	436.31	472.96	**484.92**	499.08	716.27	81.67
PH	42	all	loss	26.25	31.65	30.76	**30.32**	32.85	21.19	8.16
PH	42	*desi*	control	454.4	615.11	653.74	**670.17**	727.24	886.55	90.94
PH	42	*desi*	stress	335.11	428.59	471.49	**473.3**	489.07	716.27	81.62
PH	42	*desi*	loss	26.25	30.32	27.88	**29.38**	32.75	19.21	10.25
PH	42	*kabuli*	control	597.33	671.05	697.48	**721.63**	753.39	908.82	80.31
PH	42	*kabuli*	stress	387.29	437.23	472.96	**496.53**	530.63	708.31	81.42
PH	42	*kabuli*	loss	35.16	34.84	32.19	**31.19**	29.57	22.06	-1.38
r2g	7	all	control	0.018	0.025	0.027	**0.028**	0.03	0.043	0.005
r2g	7	all	stress	0.016	0.025	0.028	**0.028**	0.03	0.041	0.005
r2g	7	all	loss	11.11	0.00	-3.70	**0.00**	0.00	4.65	0.00

r2g	7	*desi*	control	0.024	0.026	0.029	**0.03**	0.032	0.043	0.005
r2g	7	*desi*	stress	0.019	0.025	0.028	**0.029**	0.032	0.041	0.005
r2g	7	*desi*	loss	20.83	3.85	3.45	**3.33**	0.00	4.65	0.00
r2g	7	*kabuli*	control	0.018	0.023	0.025	**0.026**	0.028	0.035	0.004
r2g	7	*kabuli*	stress	0.016	0.024	0.027	**0.026**	0.029	0.035	0.004
r2g	7	*kabuli*	loss	11.11	-4.35	-8.00	**0.00**	-3.57	0.00	0.00
r2g	28	all	control	0.003	0.004	0.005	**0.005**	0.006	0.011	0.002
r2g	28	all	stress	0.008	0.011	0.013	**0.013**	0.015	0.021	0.003
r2g	28	all	loss	-166.67	-175.00	-160.00	**-160.00**	-150.00	-90.91	-50.00
r2g	28	*desi*	control	0.003	0.005	0.006	**0.006**	0.007	0.011	0.002
r2g	28	*desi*	stress	0.008	0.012	0.015	**0.014**	0.016	0.021	0.003
r2g	28	*desi*	loss	-166.67	-140.00	-150.00	**-133.33**	-128.57	-90.91	-50.00
r2g	28	*kabuli*	control	0.003	0.004	0.005	**0.005**	0.005	0.006	0.001
r2g	28	*kabuli*	stress	0.008	0.011	0.012	**0.012**	0.014	0.019	0.003
r2g	28	*kabuli*	loss	-166.67	-175.00	-140.00	**-140.00**	-180.00	-216.67	-200.00
r2g	42	all	control	0.002	0.003	0.004	**0.004**	0.004	0.006	0.001
r2g	42	all	stress	0.003	0.005	0.005	**0.006**	0.007	0.011	0.002
r2g	42	all	loss	-50.00	-66.67	-25.00	**-50.00**	-75.00	-83.33	-100.00
r2g	42	*desi*	control	0.002	0.003	0.004	**0.004**	0.005	0.006	0.001
r2g	42	*desi*	stress	0.003	0.005	0.006	**0.006**	0.007	0.011	0.002
r2g	42	*desi*	loss	-50.00	-66.67	-50.00	**-50.00**	-40.00	-83.33	-100.00
r2g	42	*kabuli*	control	0.002	0.003	0.003	**0.003**	0.004	0.004	0.001
r2g	42	*kabuli*	stress	0.004	0.005	0.005	**0.005**	0.005	0.011	0.001
r2g	42	*kabuli*	loss	-100.00	-66.67	-66.67	**-66.67**	-25.00	-175.00	0.00
y2g	7	all	control	0.063	0.074	0.079	**0.083**	0.089	0.136	0.013
y2g	7	all	stress	0.051	0.072	0.082	**0.08**	0.088	0.112	0.011
y2g	7	all	loss	19.05	2.70	-3.80	**3.61**	1.12	17.65	15.38
y2g	7	*desi*	control	0.071	0.078	0.086	**0.088**	0.095	0.136	0.013
y2g	7	*desi*	stress	0.067	0.077	0.084	**0.084**	0.09	0.112	0.01
y2g	7	*desi*	loss	5.63	1.28	2.33	**4.55**	5.26	17.65	23.08

y2g	7	*kabuli*	control	0.063	0.071	0.075	**0.077**	0.083	0.103	0.01
y2g	7	*kabuli*	stress	0.051	0.07	0.077	**0.077**	0.088	0.093	0.011
y2g	7	*kabuli*	loss	19.05	1.41	-2.67	**0.00**	-6.02	9.71	-10.00
y2g	28	all	control	0.019	0.026	0.028	**0.029**	0.032	0.047	0.006
y2g	28	all	stress	0.042	0.06	0.069	**0.069**	0.078	0.115	0.015
y2g	28	all	loss	-121.05	-130.77	-146.43	**-137.93**	-143.75	-144.68	-150.00
y2g	28	*desi*	control	0.023	0.028	0.032	**0.032**	0.036	0.047	0.006
y2g	28	*desi*	stress	0.042	0.066	0.072	**0.073**	0.083	0.115	0.017
y2g	28	*desi*	loss	-82.61	-135.71	-125.00	**-128.13**	-130.56	-144.68	-183.33
y2g	28	*kabuli*	control	0.019	0.024	0.026	**0.026**	0.028	0.036	0.004
y2g	28	*kabuli*	stress	0.045	0.057	0.065	**0.065**	0.074	0.092	0.011
y2g	28	*kabuli*	loss	-136.84	-137.50	-150.00	**-150.00**	-164.29	-155.56	-175.00
y2g	42	all	control	0.022	0.027	0.029	**0.029**	0.031	0.042	0.004
y2g	42	all	stress	0.024	0.033	0.037	**0.038**	0.042	0.064	0.008
y2g	42	all	loss	-9.09	-22.22	-27.59	**-31.03**	-35.48	-52.38	-100.00
y2g	42	*desi*	control	0.022	0.028	0.031	**0.031**	0.033	0.042	0.005
y2g	42	*desi*	stress	0.027	0.034	0.04	**0.04**	0.046	0.064	0.009
y2g	42	*desi*	loss	-22.73	-21.43	-29.03	**-29.03**	-39.39	-52.38	-80.00
y2g	42	*kabuli*	control	0.022	0.026	0.028	**0.028**	0.029	0.031	0.003
y2g	42	*kabuli*	stress	0.024	0.032	0.035	**0.036**	0.041	0.047	0.005
y2g	42	*kabuli*	loss	-9.09	-23.08	-25.00	**-28.57**	-41.38	-51.61	-66.67

Table S3: Descriptive statistics of fresh and dry weight.

Based on BLUEs across both experiments and all 60 genotypes. Fresh and dry weight in gram; Loss [%] = (1-(stress/control))*100. q1= Quantile 25%; q3= Quantile 75%; sd=standard deviation.

	**control**		**stress**		**loss**	
	**fresh**	**dry**	**fresh**	**dry**	**fresh**	**dry**
**min**	35.33	5.43	14.05	1.93	60.23	64.46
**q1**	44.42	7.18	19.31	2.92	56.53	59.33
**median**	55.35	8.8	21.48	3.23	61.19	63.30
**mean**	53.99	8.84	21.48	3.25	60.21	63.24
**q3**	61.97	10.55	23.77	3.61	61.64	65.78
**max**	77.28	12.58	27.83	4.23	63.99	66.38

Table S4: Descriptive statistics of RGR.

Based on BLUEs across both experiments and all 60 genotypes.

RGR [voxel /DAT] = relative growth rate = $(\ln(EB_i)-\ln(EB_{i-1}))/(DAT_i-DAT_{i-1})$

	stress		control	
DAT	EB	RGR	EB	RGR
1	1.09	NA	1.09	NA
2	2.18	0.69	2.17	0.69
3	2.62	0.18	2.60	0.18
4	3.18	0.20	3.20	0.21
5	3.61	0.13	3.57	0.11
6	3.94	0.09	3.87	0.08
7	4.45	0.12	4.42	0.13
8	5.74	0.25	5.79	0.27
9	6.75	0.16	6.82	0.16
10	7.61	0.12	7.95	0.15
11	8.75	0.14	9.37	0.16
12	9.86	0.12	10.72	0.13
13	10.85	0.10	12.74	0.17
14	11.81	0.08	14.87	0.15
15	12.76	0.08	17.00	0.13
16	13.11	0.03	18.69	0.09
17	14.12	0.07	21.98	0.16
18	15.00	0.06	26.19	0.18
19	15.94	0.06	30.69	0.16
20	16.47	0.03	35.35	0.14
21	17.29	0.05	40.15	0.13
22	16.74	-0.03	43.27	0.07
23	17.52	0.05	50.51	0.15
24	17.37	-0.01	55.31	0.09
25	17.50	0.01	60.55	0.09

26	18.67	0.06	70.25	0.15
27	17.55	-0.06	75.68	0.07
28	18.55	0.06	85.15	0.12
29	17.53	-0.06	88.83	0.04
30	21.30	0.19	98.38	0.10
31	23.55	0.10	108.75	0.10
32	25.72	0.09	117.26	0.08
33	28.20	0.09	129.29	0.10
34	30.34	0.07	134.51	0.04
35	33.49	0.10	145.15	0.08
36	37.35	0.11	156.21	0.07
37	41.42	0.10	164.74	0.05
38	45.51	0.09	178.28	0.08
39	49.46	0.08	190.28	0.07
40	54.58	0.10	204.61	0.07
41	59.90	0.09	208.08	0.02
42	63.56	0.06	214.70	0.03

Table S5: Descriptive statistics of WUE for drought phases and chickpea types.

Based on BLUEs across both experiments and all 60 genotypes or *desi* and *kabuli*; q1= Quantile 25%; q3= Quantile 75%; sd=standard deviation.

**chickpea**	**phase**	**treatment**	**min**	**q1**	**median**	**mean**	**q3**	**max**	**sd**
all	**DA**	control	0.019	0.033	0.037	**0.038**	0.042	0.058	0.008
		stress	0.025	0.035	0.039	**0.039**	0.044	0.061	0.007
all	**DR**	control	0.015	0.032	0.039	**0.040**	0.046	0.068	0.011
		stress	0.012	0.022	0.026	**0.026**	0.030	0.043	0.007
all	**DT**	control	0.022	0.029	0.033	**0.033**	0.038	0.048	0.006
		stress	0.215	7.603	11.699	**9.630**	12.831	16.765	5.455
desi	**DA**	control	0.019	0.030	0.035	**0.036**	0.041	0.052	0.008
		stress	0.025	0.032	0.036	**0.037**	0.042	0.052	0.007
kabuli	**DA**	control	0.029	0.036	0.039	**0.041**	0.045	0.058	0.007
		stress	0.029	0.038	0.041	**0.042**	0.044	0.061	0.006
desi	**DR**	control	0.015	0.031	0.038	**0.038**	0.046	0.061	0.011
		stress	0.012	0.018	0.023	**0.024**	0.028	0.038	0.007
kabuli	**DR**	control	0.025	0.035	0.041	**0.043**	0.048	0.068	0.011
		stress	0.016	0.026	0.027	**0.028**	0.030	0.043	0.005
desi	**DT**	control	0.022	0.026	0.030	**0.030**	0.034	0.041	0.005
		stress	0.587	10.829	12.025	**11.495**	13.195	15.713	3.466
kabuli	**DT**	control	0.025	0.033	0.036	**0.036**	0.039	0.048	0.005
		stress	0.215	0.595	10.943	**7.765**	12.494	16.765	6.428

Table S6: Tukey`s test of WUE in different drought phases and for desi and kabuli under drought phase.

ANOVA describes whether there was a significant influence in general of the WUE and the phase / *desi* and *kabuli*, the WUE and the two treatments, and the phase / *desi* and *kabuli* and the treatments.

	**combination**	**diff**	**p adj**	**ANOVA**
**phase**	DR-DA	-0.006	1.000	***
	DT-DA	4.833	**0.000**	***
	DT-DR	4.839	**0.000**	***
	stress-control	3.186	**0.000**	***
	DR:control-DA:control	0.002	1.000	***
	DT:control-DA:control	-0.005	1.000	***
	DA:stress-DA:control	0.001	1.000	***
	DR:stress-DA:control	-0.012	1.000	***
	DT:stress-DA:control	9.592	**0.000**	***
	DT:control-DR:control	-0.007	1.000	***
	DA:stress-DR:control	-0.001	1.000	***
	DR:stress-DR:control	-0.014	1.000	***
	DT:stress-DR:control	9.590	**0.000**	***
	DA:stress-DT:control	0.006	1.000	***
	DR:stress-DT:control	-0.007	1.000	***
	DT:stress-DT:control	9.597	**0.000**	***
	DR:stress-DA:stress	-0.013	1.000	***
	DT:stress-DA:stress	9.591	**0.000**	***
	DT:stress-DR:stress	9.604	**0.000**	***
	DT:stress-DR:stress	9.604	**0.000**	***
**DT**	***kabuli- desi***	-1.797	**0.009**	**
	stress-control	9.612	**0.000**	***
	kabuli:control- *desi*:control	0.005	1.000	**
	desi:stress- *desi*:control	11.464	**0.000**	**
	kabuli:stress- *desi:*control	7.735	**0.000**	**
	desi:stress- *kabuli*:control	11.459	**0.000**	**
	kabuli:stress- *kabuli*:control	7.729	**0.000**	**
	kabuli:stress- *desi*:stress	-3.730	**0.001**	**
**DR**	***kabuli - desi***	0.005	**0.004**	**
	stress-control	-0.014	**0.000**	***
	kabuli:control- *desi*:control	0.004	0.204	
	desi:stress- *desi*:control	-0.015	**0.000**	
	kabuli:stress- *desi*:control	-0.010	**0.000**	
	desi:stress- *kabuli*:control	-0.019	**0.000**	
	kabuli:stress- *kabuli*:control	-0.014	**0.000**	

	kabuli:stress- *desi*:stress	0.005	0.146	
**DA**	***kabuli - desi***	0.005	**0.000**	***
	stress-control	0.001	0.313	
	kabuli:control- *desi*:control	0.005	**0.034**	
	desi:stress- *desi*:control	0.002	0.841	
	kabuli:stress- *desi*:control	0.006	**0.005**	
	desi:stress- *kabuli*:control	-0.004	0.218	
	kabuli:stress- *kabuli*:control	0.001	0.931	
	kabuli:stress- *desi*:stress	0.005	0.056	

Table S7: Descriptive statistics of Chlorophyll Fluorescence traits. Based on BLUEs across both experiments and all genotypes or *desi* and *kabuli* genotypes.

Loss [%] = (1-(stress/control))*100. q1= Quantile 25%; q3= Quantile 75%; sd=standard deviation. ΦPSII = operating efficiency of photosystem II under high and low light condition. Ratio= ΦPSIIl / ΦPSII h

ΦPSII	DAT	chickpea	treatment	min	q1	median	mean	q3	max	sd
high	6	all	control	0.41	0.46	0.47	**0.47**	0.48	0.5	0.02
high	6	all	stress	0.42	0.47	0.47	**0.47**	0.48	0.51	0.02
high	6	all	loss	-2.44	-2.17	0.00	**0.00**	0.00	-2.00	0.00
high	6	*desi*	control	0.42	0.46	0.47	**0.47**	0.48	0.49	0.02
high	6	*desi*	stress	0.43	0.46	0.47	**0.47**	0.49	0.5	0.02
high	6	*desi*	loss	-2.38	0.00	0.00	**0.00**	-2.08	-2.04	0.00
high	6	*kabuli*	control	0.41	0.46	0.47	**0.47**	0.48	0.5	0.02
high	6	*kabuli*	stress	0.42	0.47	0.47	**0.47**	0.48	0.51	0.02
high	6	*kabuli*	loss	-2.44	-2.17	0.00	**0.00**	0.00	-2.00	0.00
high	13	all	control	0.39	0.43	0.44	**0.44**	0.46	0.48	0.02
high	13	all	stress	0.4	0.44	0.45	**0.44**	0.45	0.48	0.02
high	13	all	loss	-2.56	-2.33	-2.27	**0.00**	2.17	0.00	0.00
high	13	*desi*	control	0.39	0.44	0.44	**0.44**	0.45	0.48	0.02
high	13	*desi*	stress	0.4	0.44	0.45	**0.45**	0.46	0.48	0.02
high	13	*desi*	loss	-2.56	0.00	-2.27	**-2.27**	-2.22	0.00	0.00
high	13	*kabuli*	control	0.4	0.43	0.44	**0.44**	0.46	0.48	0.02
high	13	*kabuli*	stress	0.4	0.44	0.45	**0.44**	0.45	0.46	0.02
high	13	*kabuli*	loss	0.00	-2.33	-2.27	**0.00**	2.17	4.17	0.00
high	20	all	control	0.36	0.42	0.43	**0.43**	0.45	0.47	0.03
high	20	all	stress	0.34	0.39	0.4	**0.4**	0.42	0.44	0.02
high	20	all	loss	5.56	7.14	6.98	**6.98**	6.67	6.38	33.33
high	20	*desi*	control	0.36	0.42	0.44	**0.43**	0.45	0.47	0.03
high	20	*desi*	stress	0.36	0.39	0.4	**0.4**	0.42	0.44	0.02

high	20	*desi*	loss	0.00	7.14	9.09	**6.98**	6.67	6.38	33.33
high	20	*kabuli*	control	0.37	0.41	0.43	**0.43**	0.45	0.47	0.03
high	20	*kabuli*	stress	0.34	0.38	0.4	**0.4**	0.41	0.43	0.02
high	20	*kabuli*	loss	8.11	7.32	6.98	**6.98**	8.89	8.51	33.33
high	27	all	control	0.38	0.42	0.44	**0.44**	0.46	0.49	0.03
high	27	all	stress	0.27	0.34	0.35	**0.35**	0.38	0.41	0.03
high	27	all	loss	28.95	19.05	20.45	**20.45**	17.39	16.33	0.00
high	27	*desi*	control	0.38	0.42	0.44	**0.44**	0.46	0.49	0.03
high	27	*desi*	stress	0.31	0.34	0.35	**0.36**	0.38	0.4	0.03
high	27	*desi*	loss	18.42	19.05	20.45	**18.18**	17.39	18.37	0.00
high	27	*kabuli*	control	0.39	0.42	0.43	**0.43**	0.45	0.48	0.02
high	27	*kabuli*	stress	0.27	0.33	0.35	**0.35**	0.38	0.41	0.03
high	27	*kabuli*	loss	30.77	21.43	18.60	**18.60**	15.56	14.58	-50.00
high	34	all	control	0.35	0.41	0.43	**0.43**	0.45	0.49	0.03
high	34	all	stress	0.39	0.42	0.43	**0.43**	0.44	0.48	0.02
high	34	all	loss	-11.43	-2.44	0.00	**0.00**	2.22	2.04	33.33
high	34	*desi*	control	0.35	0.41	0.43	**0.43**	0.46	0.49	0.03
high	34	*desi*	stress	0.41	0.43	0.44	**0.44**	0.45	0.48	0.02
high	34	*desi*	loss	-17.14	-4.88	-2.33	**-2.33**	2.17	2.04	33.33
high	34	*kabuli*	control	0.36	0.41	0.42	**0.42**	0.45	0.48	0.03
high	34	*kabuli*	stress	0.39	0.41	0.43	**0.43**	0.44	0.47	0.02
high	34	*kabuli*	loss	-8.33	0.00	-2.38	**-2.38**	2.22	2.08	33.33
low	6	all	control	0.6	0.62	0.63	**0.63**	0.63	0.65	0.01
low	6	all	stress	0.6	0.62	0.62	**0.62**	0.63	0.65	0.01
low	6	all	loss	0.00	0.00	1.59	**1.59**	0.00	0.00	0.00
low	13	all	control	0.61	0.63	0.63	**0.64**	0.64	0.66	0.01
low	13	all	stress	0.62	0.64	0.64	**0.64**	0.65	0.67	0.01
low	13	all	loss	-1.64	-1.59	-1.59	**0.00**	-1.56	-1.52	0.00
low	20	all	control	0.62	0.64	0.65	**0.65**	0.65	0.67	0.01
low	20	all	stress	0.57	0.61	0.62	**0.62**	0.63	0.66	0.02

low	20	all	loss	8.06	4.69	4.62	**4.62**	3.08	1.49	-100.00
low	27	all	control	0.62	0.64	0.65	**0.65**	0.66	0.67	0.01
low	27	all	stress	0.51	0.55	0.57	**0.57**	0.58	0.61	0.02
low	27	all	loss	17.74	14.06	12.31	**12.31**	12.12	8.96	-100.00
low	34	all	control	0.61	0.63	0.64	**0.64**	0.65	0.66	0.01
low	34	all	stress	0.61	0.63	0.63	**0.63**	0.64	0.65	0.01
low	34	all	loss	0.00	0.00	1.56	**1.56**	1.54	1.52	0.00
ratio	6	all	control	1.24	1.31	1.34	**1.34**	1.36	1.53	0.06
ratio	6	all	stress	1.21	1.3	1.33	**1.33**	1.35	1.52	0.06
ratio	6	all	loss	2.42	0.76	0.75	**0.75**	0.74	0.65	0.00
ratio	13	all	control	1.31	1.39	1.44	**1.44**	1.48	1.71	0.08
ratio	13	all	stress	1.33	1.41	1.44	**1.45**	1.47	1.66	0.07
ratio	13	all	loss	-1.53	-1.44	0.00	**-0.69**	0.68	2.92	12.50
ratio	20	all	control	1.33	1.43	1.5	**1.52**	1.57	1.83	0.11
ratio	20	all	stress	1.41	1.51	1.56	**1.56**	1.61	1.81	0.09
ratio	20	all	loss	-6.02	-5.59	-4.00	**-2.63**	-2.55	1.09	18.18
ratio	27	all	control	1.27	1.41	1.49	**1.49**	1.57	1.76	0.11
ratio	27	all	stress	1.44	1.57	1.65	**1.65**	1.7	1.91	0.11
ratio	27	all	loss	-13.39	-11.35	-10.74	**-10.74**	-8.28	-8.52	0.00
ratio	34	all	control	1.27	1.41	1.48	**1.5**	1.59	1.86	0.14
ratio	34	all	stress	1.27	1.43	1.47	**1.47**	1.51	1.7	0.07
ratio	34	all	loss	0.00	-1.42	0.68	**2.00**	5.03	8.60	50.00

Table S8: Tukey's test for chlorophyll fluorescence traits

Based on BLUEs across for both experiments. ANOVA describes whether there was a significant influence of the WUE and the phase / *desi* and *kabuli*, the WUE and the two treatments, and the phase / *desi* and *kabuli* and the treatments.

ΦPSII = operating efficiency of photosystem II under high light condition.

**ΦPSII**	**combination**	**diff**	**p adj**	**ANOVA**
**high**	13-6	-0.027	**0.000**	***
	20-6	-0.055	**0.000**	***
	27-6	-0.075	**0.000**	***
	34-6	-0.039	**0.000**	***
	20-13	-0.028	**0.000**	***
	27-13	-0.048	**0.000**	***
	34-13	-0.013	**0.000**	***
	27-20	-0.020	**0.000**	***
	34-20	0.016	**0.000**	***
	34-27	0.035	**0.000**	***
	stress-control	-0.021	**0.000**	***
	13:control-6:control	-0.026	**0.000**	***
	20:control-6:control	-0.039	**0.000**	***
	27:control-6:control	-0.031	**0.000**	***
	34:control-6:control	-0.039	**0.000**	***
	6:stress-6:control	0.004	0.994	***
	13:stress-6:control	-0.024	**0.000**	***
	20:stress-6:control	-0.068	**0.000**	***
	27:stress-6:control	-0.115	**0.000**	***
	34:stress-6:control	-0.036	**0.000**	***
	20:control-13:control	-0.013	0.085	***
	27:control-13:control	-0.005	0.967	***
	34:control-13:control	-0.013	0.058	***
	6:stress-13:control	0.030	**0.000**	***
	13:stress-13:control	0.002	1.000	***
	20:stress-13:control	-0.042	**0.000**	***
	27:stress-13:control	-0.089	**0.000**	***
	34:stress-13:control	-0.010	0.365	***
	27:control-20:control	0.007	0.764	***
	34:control-20:control	-0.001	1.000	***
	6:stress-20:control	0.043	**0.000**	***
	13:stress-20:control	0.015	**0.024**	***
	20:stress-20:control	-0.029	**0.000**	***
	27:stress-20:control	-0.076	**0.000**	***
	34:stress-20:control	0.003	1.000	***
	34:control-27:control	-0.008	0.677	***
	6:stress-27:control	0.035	**0.000**	***

	13:stress-27:control	0.007	0.816	***
	20:stress-27:control	-0.037	**0.000**	***
	27:stress-27:control	-0.083	**0.000**	***
	34:stress-27:control	-0.005	0.984	***
	6:stress-34:control	0.043	**0.000**	***
	13:stress-34:control	0.015	**0.015**	***
	20:stress-34:control	-0.029	**0.000**	***
	27:stress-34:control	-0.075	**0.000**	***
	34:stress-34:control	0.003	0.999	***
	13:stress-6:stress	-0.028	**0.000**	***
	20:stress-6:stress	-0.072	**0.000**	***
	27:stress-6:stress	-0.119	**0.000**	***
	34:stress-6:stress	-0.040	**0.000**	***
	20:stress-13:stress	-0.044	**0.000**	***
	27:stress-13:stress	-0.091	**0.000**	***
	34:stress-13:stress	-0.012	0.150	***
	27:stress-20:stress	-0.047	**0.000**	***
	34:stress-20:stress	0.032	**0.000**	***
	34:stress-27:stress	0.079	**0.000**	***
**DAT 6**	***kabuli-desi***	-0.005	0.112	
	stress-control	-0.021	**0.000**	***
**DAT 13**	***kabuli-desi***	-0.001	0.766	
	stress-control	0.002	0.601	
**DAT 20**	***kabuli-desi***	-0.007	0.137	
	stress-control	-0.029	**0.000**	***
**DAT 27**	***kabuli-desi***	-0.009	0.087	
	stress-control	-0.083	**0.000**	***
**DAT 34**	***kabuli-desi***	-0.011	**0.026**	*
	stress-control	0.003	0.485	

Table S9: Correlations of EB with further image-derived traits during the period of DA, DR and DT.

EB [voxel e-05]; PH [mm]; MCV [hue]; R2G [%]; Y2G [%].

The coefficient of correlation *r* and the level of significance *p-value* (*** > 0.001) are displayed. Dark red field means a high positive coefficient of correlation. A dark blue field means a high negative coefficient of correlation.

Based on BLUEs across both experiments and all 60 genotypes. DA=drought adaptability: DAT 8-42; DR=drought recovery DAT 29-42; DT=drought tolerance DAT 8-28.

	**DA**				**DR**				**DT**			
	control		stress		control		stress		control		stress	
Correlation of EB with	*r*	*p-value*	*r*	*p-value*	*r*	*p-value*	*r*	*p-value*	*r*	*p-value*	*r*	*p-value*
PH	0.87	***	0.71	***	0.7	***	0.56	***	0.83	***	0.58	***
MCV	-0.64	***	-0.61	***	-0.47	***	-0.72	***	-0.54	***	0.53	***
R2G	-0.69	***	-0.74	***	-0.54	***	-0.73	***	-0.76	***	-0.76	***
Y2G	-0.61	***	-0.54	***	-0.0052		-0.74	***	-0.74	***	-0.33	***

Table S10: Correlation between ΦPSIIh and image-derived traits.

Based on both treatments and BLUEs across both experiments of all 60 genotypes. DAT 6 = 1 day before drought, DAT 13 = 7 days of drought, DAT 20 = 13 days of drought, DAT 27 = 19 days of drought, DAT 34 = 6 days of recovery. ϕPSIIh = operating efficiency of photosystem II under high light conditions.

Dark red field means a high positive coefficient of correlation *r*. A dark blue field means a high negative coefficient of correlation *r*. Significant correlations with a *p-value* < 0.05 are highlighted in green.

	DAT 6		DAT 13		DAT 20		DAT 27		DAT 34	
	r	*p-value*	*r*	*p-value*	*r*	*p-value*	*r*	*p-value*	*r*	*p-value*
EB	0.22	0.014	0.08	0.388	0.32	0.000	0.69	0.000	-0.13	0.143
PH	-0.40	0.000	-0.52	0.000	0.02	0.855	0.55	0.000	-0.34	0.000
MCV	-0.18	0.054	0.20	0.032	-0.22	0.018	-0.62	0.000	0.31	0.001
r2g	-0.19	0.034	-0.01	0.910	-0.22	0.017	-0.70	0.000	0.11	0.233
y2g	-0.10	0.267	0.01	0.874	-0.27	0.003	-0.68	0.000	0.11	0.212

Table S11: Superior genotypes for EB and WUE as measured by the difference from the mean of the panel under drought stress compared to the control treatment.

Superior genotypes INCCP_00119, INCCP_00139, INCCP_00291 and INCCP_01917 are highlighted bold. DAT = day after transferring; DAT 28 = longest period of drought stress; DAT 42 = longest period of recovery; WUE = water use efficiency; phase: DA= drought adaptability DAT 8-42; DT= drought tolerance DAT 8-28 and DR= drought recovery DAT 29-42.

Genotype	WUE of drought phase	DAT	Difference control	Difference stress	DK	Country	Geographic Area	Biological Status
INCCP_00004	-	28	11.62	0.89	*kabuli*	ALB	Balkanic Europe	landrace
INCCP_00006	-	28	32.8	0.45	*desi*	BGR	Balkanic Europe	breeding material
INCCP_00009	-	28	26.44	1.03	*desi*	EGY	Nord Africa	landrace
INCCP_00015	-	28	39.21	1.6	*desi*	GRC	Balkanic Europe	landrace
INCCP_00017	DT	-	0.004	9.043	*desi*	GRC	Balkanic Europe	landrace
INCCP_00017	DR	-	0.007	0.003	*desi*	GRC	Balkanic Europe	landrace
INCCP_00017	DA	-	0.007	0.003	*desi*	GRC	Balkanic Europe	landrace
INCCP_00049	-	28	0.42	0.77	*kabuli*	ITA	Italy	landrace
INCCP_00049	-	42	27.99	1.26	*kabuli*	ITA	Italy	landrace
INCCP_00049	DR	-	0.003	0.001	*kabuli*	ITA	Italy	landrace
INCCP_00049	DA	-	0.003	0.001	*kabuli*	ITA	Italy	landrace
INCCP_00102	DR	-	0.001	0.004	*kabuli*	TUN	Nord Africa	landrace
INCCP_00102	DA	-	0.001	0.004	*kabuli*	TUN	Nord Africa	landrace
INCCP_00103	-	42	13.01	15.18	*desi*	TUN	Nord Africa	landrace
INCCP_00103	DR	-	0.005	0.007	*desi*	TUN	Nord Africa	landrace
INCCP_00103	DA	-	0.005	0.007	*desi*	TUN	Nord Africa	landrace
**INCCP_00119**	-	28	7.74	0.89	*desi*	TUR	Turkey	landrace
**INCCP_00119**	-	42	44.94	13.03	*desi*	TUR	Turkey	landrace
**INCCP_00119**	DT	-	0.003	8.922	*desi*	TUR	Turkey	landrace
**INCCP_00119**	DR	-	0.008	0.009	*desi*	TUR	Turkey	landrace
**INCCP_00119**	DA	-	0.008	0.009	*desi*	TUR	Turkey	landrace

**INCCP_00139**	-	28	24.43	5.86	*desi*	TJK	Asia	landrace
**INCCP_00139**	-	42	102.41	28.67	*desi*	TJK	Asia	landrace
**INCCP_00139**	DT	-	0.005	0.357	*desi*	TJK	Asia	landrace
**INCCP_00139**	DR	-	0.013	0.011	*desi*	TJK	Asia	landrace
**INCCP_00139**	DA	-	0.013	0.011	*desi*	TJK	Asia	landrace
INCCP_00154	-	28	25.85	2.05	*kabuli*	HUN	East Europe	cultivar
INCCP_00193	-	28	38.61	3.58	*desi*	AFG	Asia	landrace
INCCP_00193	-	42	103.92	25	*desi*	AFG	Asia	landrace
INCCP_00193	DR	-	0.011	0.005	*desi*	AFG	Asia	landrace
INCCP_00193	DA	-	0.011	0.005	*desi*	AFG	Asia	landrace
INCCP_00216	-	28	36.98	4.13	*desi*	MKD	Balkanic Europe	cultivar
INCCP_00216	-	42	50.48	10.34	*desi*	MKD	Balkanic Europe	cultivar
INCCP_00216	DR	-	0	0.002	*desi*	MKD	Balkanic Europe	cultivar
INCCP_00216	DA	-	0	0.002	*desi*	MKD	Balkanic Europe	cultivar
INCCP_00232	-	28	9.44	1.56	*desi*	ESP	Iberian Peninsula	cultivar
INCCP_00232	-	42	25.89	2.88	*desi*	ESP	Iberian Peninsula	cultivar
INCCP_00232	DR	-	0.002	0.002	*desi*	ESP	Iberian Peninsula	cultivar
INCCP_00232	DA	-	0.002	0.002	*desi*	ESP	Iberian Peninsula	cultivar
**INCCP_00291**	-	28	37.73	5.74	*desi*	MEX	Central America	cultivar
**INCCP_00291**	-	42	83.76	31.04	*desi*	MEX	Central America	cultivar
**INCCP_00291**	DT	-	0.007	0.096	*desi*	MEX	Central America	cultivar
**INCCP_00291**	DR	-	0.008	0.009	*desi*	MEX	Central America	cultivar
**INCCP_00291**	DA	-	0.008	0.009	*desi*	MEX	Central America	cultivar
INCCP_00295	-	28	17.31	1.41	*desi*	PAK	Indian Continent	cultivar
INCCP_00295	-	42	67.04	17.5	*desi*	PAK	Indian Continent	cultivar
INCCP_00295	DR	-	0.011	0.007	*desi*	PAK	Indian Continent	cultivar
INCCP_00295	DA	-	0.011	0.007	*desi*	PAK	Indian Continent	cultivar
INCCP_00372	-	28	22.14	3.73	*desi*	TUR	Turkey	landrace
INCCP_00372	-	42	40.13	16.53	*desi*	TUR	Turkey	landrace
INCCP_00372	DR	-	0.004	0.005	*desi*	TUR	Turkey	landrace

INCCP_00372	DA	-	0.004	0.005	*desi*	TUR	Turkey	landrace
INCCP_00429	-	28	36.17	0.77	*desi*	PAK	Indian Continent	landrace
INCCP_00429	-	42	81.08	12.51	*desi*	PAK	Indian Continent	landrace
INCCP_00429	DR	-	0.01	0.005	*desi*	PAK	Indian Continent	landrace
INCCP_00429	DA	-	0.01	0.005	*desi*	PAK	Indian Continent	landrace
INCCP_00438	-	28	23.29	3.57	*desi*	CHN	Asia	landrace
INCCP_00438	-	42	45.28	5.85	*desi*	CHN	Asia	landrace
INCCP_00438	DR	-	0.001	0.004	*desi*	CHN	Asia	landrace
INCCP_00438	DA	-	0.001	0.004	*desi*	CHN	Asia	landrace
INCCP_00447	-	28	19.21	3.39	*desi*	ETH	Ethiopia	landrace
INCCP_00447	-	42	84.73	28.53	*desi*	ETH	Ethiopia	landrace
INCCP_00447	DR	-	0.01	0.006	*desi*	ETH	Ethiopia	landrace
INCCP_00447	DA	-	0.01	0.006	*desi*	ETH	Ethiopia	landrace
INCCP_00466	-	28	38.24	3.38	*desi*	BGD	Indian Continent	cultivar
INCCP_00466	-	42	128.94	30.87	*desi*	BGD	Indian Continent	cultivar
INCCP_00466	DR	-	0.019	0.014	*desi*	BGD	Indian Continent	cultivar
INCCP_00466	DA	-	0.019	0.014	*desi*	BGD	Indian Continent	cultivar
INCCP_00470	-	28	35.95	4.58	*desi*	UZB	Asia	domesticated material
INCCP_00470	-	42	44.83	14.63	*desi*	UZB	Asia	domesticated material
INCCP_00470	DT	-	0.008	0.368	*desi*	UZB	Asia	domesticated material
INCCP_00470	DR	-	0.003	0.008	*desi*	UZB	Asia	domesticated material
INCCP_00470	DA	-	0.003	0.008	*desi*	UZB	Asia	domesticated material
INCCP_00480	-	28	25.85	3.57	*desi*	MDA	Balkanic Europe	landrace
INCCP_00480	-	42	68.3	15.88	*desi*	MDA	Balkanic Europe	landrace
INCCP_00486	-	28	5.46	3.8	*desi*	AUS	Others	cultivar
INCCP_00486	-	42	52.97	14.57	*desi*	AUS	Others	cultivar
INCCP_00486	DR	-	0.005	0.01	*desi*	AUS	Others	cultivar
INCCP_00486	DA	-	0.005	0.01	*desi*	AUS	Others	cultivar
INCCP_00508	-	28	6.83	2.06	*desi*	SYR	Middle East	breeding material
INCCP_00508	-	42	20.62	18.04	*desi*	SYR	Middle East	breeding material

INCCP_00508	DR	-	0.004	0.01	*desi*	SYR	Middle East	breeding material
INCCP_00508	DA	-	0.004	0.01	*desi*	SYR	Middle East	breeding material
INCCP_00512	DR	-	0.007	0.002	*kabuli*	ESP	Iberian Peninsula	breeding material
INCCP_00512	DA	-	0.007	0.002	*kabuli*	ESP	Iberian Peninsula	breeding material
INCCP_00573	-	28	31.9	4.33	*desi*	IND	Indian Continent	landrace
INCCP_00573	-	42	81.01	35.38	*desi*	IND	Indian Continent	landrace
INCCP_00573	DR	-	0.011	0.012	*desi*	IND	Indian Continent	landrace
INCCP_00573	DA	-	0.011	0.012	*desi*	IND	Indian Continent	landrace
INCCP_01429	DR	-	0.007	0.003	*kabuli*	CYP	Middle East	landrace
INCCP_01429	DA	-	0.007	0.003	*kabuli*	CYP	Middle East	landrace
INCCP_01461	DR	-	0.001	0.001	*kabuli*	TUR	Turkey	landrace
INCCP_01461	DA	-	0.001	0.001	*kabuli*	TUR	Turkey	landrace
INCCP_01615	-	28	24.52	2.47	*kabuli*	SYR	Middle East	landrace
INCCP_01615	-	42	61.86	10.28	*kabuli*	SYR	Middle East	landrace
INCCP_01615	DR	-	0.009	0.006	*kabuli*	SYR	Middle East	landrace
INCCP_01615	DA	-	0.009	0.006	*kabuli*	SYR	Middle East	landrace
INCCP_01867	DR	-	0.005	0.003	*kabuli*	KAZ	Asia	landrace
INCCP_01867	DA	-	0.005	0.003	*kabuli*	KAZ	Asia	landrace
**INCCP_01917**	-	28	13.48	0.91	*kabuli*	PRT	Iberian Peninsola	landrace
**INCCP_01917**	-	42	25.49	3.32	*kabuli*	PRT	Iberian Peninsola	landrace
**INCCP_01917**	DT	-	0.003	9.04	*kabuli*	PRT	Iberian Peninsola	landrace
**INCCP_01917**	DR	-	0.002	0.003	*kabuli*	PRT	Iberian Peninsola	landrace
**INCCP_01917**	DA	-	0.002	0.003	*kabuli*	PRT	Iberian Peninsola	landrace

Table S12: Description of Plant Material.

Genotype	DOI	Line Code UNIVPM	Synonym1	Synonym2	Type Of Chickpea	Country	Geographic Area	Biological Status	Donor Institute
INCCP_00004	10.18730/Z8H4J	AN_Ca_0005	CIC 235	95469	*kabuli*	ALB	Balkanic Europe	landrace	IPK
INCCP_00006	10.18730/Z8HN=	AN_Ca_0009	CIC 63	44726	*desi*	BGR	Balkanic Europe	breeding material	IPK
INCCP_00009	10.18730/Z8FF2	AN_Ca_0015	CIC 118	44780	*desi*	EGY	Nord Africa	landrace	IPK
INCCP_00015	10.18730/Z8GJ0	AN_Ca_0024	CIC 18	44681	*desi*	GRC	Balkanic Europe	landrace	IPK
INCCP_00017	10.18730/Z8GWA	AN_Ca_0026	CIC 21	44684	*desi*	GRC	Balkanic Europe	landrace	IPK
INCCP_00034	10.18730/Z8FG3	AN_Ca_0053	CIC 124	44786	*desi*	ITA	Italy	landrace	IPK
INCCP_00049	10.18730/Z8JNY	AN_Ca_0077	CIC 91	78711	*kabuli*	ITA	Italy	landrace	IPK
INCCP_00078	10.18730/Z8G8V	AN_Ca_0124	CIC 160	79870	*kabuli*	ITA	Italy	landrace	IPK
INCCP_00100	10.18730/Z8JJV	AN_Ca_0169	CIC 84	44747	*kabuli*	SVK	East Europe	landrace	IPK
INCCP_00102	10.18730/Z8GV9	AN_Ca_0172	CIC 203	55301	*kabuli*	TUN	Nord Africa	landrace	IPK
INCCP_00103	10.18730/Z8H5K	AN_Ca_0175	CIC 237	95476	*desi*	TUN	Nord Africa	landrace	IPK
INCCP_00119	10.18730/Z8JHT	AN_Ca_0197	CIC 8	44671	*desi*	TUR	Turkey	landrace	IPK
INCCP_00139	10.18730/ZACT4	AN_Ca_0224	USSR-05-03-BD	W6 3498	*desi*	TJK	Asia	landrace	USDA
INCCP_00149	10.18730/ZA0ST	AN_Ca_0238	OBRAZTZOV CHIFLIK 1	W6 10046	*kabuli*	BGR	Balkanic Europe	landrace	USDA
INCCP_00154	10.18730/ZA10~	AN_Ca_0243	BEKESCSABAI 2	W6 11071	*kabuli*	HUN	East Europe	cultivar	USDA
INCCP_00159	10.18730/ZA151	AN_Ca_0248	1204/86	W6 11077	*desi*	FRA	West Europe	landrace	USDA
INCCP_00160	10.18730/ZA162	AN_Ca_0249	1658/85	W6 11078	*kabuli*	ROU	Balkanic Europe	landrace	USDA
INCCP_00161	10.18730/ZA173	AN_Ca_0250	125/84	W6 11079	*desi*	HUN	East Europe	landrace	USDA
INCCP_00163	10.18730/ZA195	AN_Ca_0252	417	W6 11081	*desi*	DEU	West Europe	landrace	USDA
INCCP_00165	10.18730/ZA1B7	AN_Ca_0254	Krokhmal #2	W6 11343	*kabuli*	UKR	East Europe	cultivar	USDA
INCCP_00166	10.18730/ZA1C8	AN_Ca_0255	Krokhmal #3	W6 11344	*desi*	UKR	East Europe	cultivar	USDA
INCCP_00190	10.18730/ZA24*	AN_Ca_0287	ILC 191	W6 14954	*kabuli*	RUS	Asia	landrace	USDA
INCCP_00193	10.18730/Z8MGF	AN_Ca_0290	NO. 12620	PI 207470	*desi*	AFG	Asia	landrace	USDA
INCCP_00216	10.18730/Z8N71	AN_Ca_0315	RUDNICKI	PI 370420	*desi*	MKD	Balkanic Europe	cultivar	USDA
INCCP_00219	10.18730/Z8NA4	AN_Ca_0318	KLISURSKI	PI 379220	*desi*	MKD	Balkanic Europe	cultivar	USDA
INCCP_00230	10.18730/Z8NNF	AN_Ca_0330	PEDROSILLANO	PI 533672	*kabuli*	ESP	Iberian Peninsula	cultivar	USDA

INCCP_00232	10.18730/Z8NQH	AN_Ca_0334	NEGRO VICOS	PI 533676	*desi*	ESP	Iberian Peninsula	cultivar	USDA
INCCP_00251	10.18730/Z8PAU	AN_Ca_0365	CALIA	PI 572508	*kabuli*	ITA	Italy	cultivar	USDA
INCCP_00291	10.18730/Z8QJ2	AN_Ca_0410	PORQUERO NEGRO	PI 577014	*desi*	MEX	Central America	cultivar	USDA
INCCP_00295	10.18730/Z8QP6	AN_Ca_0418	CM1913	PI 577022	*desi*	PAK	Indian continent	cultivar	USDA
INCCP_00298	10.18730/Z8QS9	AN_Ca_0421	CM-1	PI 577026	*desi*	PAK	Indian continent	cultivar	USDA
INCCP_00372	10.18730/Z8RFZ	AN_Ca_0501	300685-0703D	PI 595967	*desi*	TUR	Turkey	landrace	USDA
INCCP_00429	10.18730/ZA3YG	AN_Ca_0562	WKP 88-57	W6 17612	*desi*	PAK	Indian continent	landrace	USDA
INCCP_00438	10.18730/ZA2G7	AN_Ca_0571	W6 17431	W6 17431	*desi*	CHN	Asia	landrace	USDA
INCCP_00447	10.18730/ZA2SG	AN_Ca_0580	ICC 8578	W6 17447	*desi*	ETH	Ethiopia	landrace	USDA
INCCP_00466	10.18730/Z8TX=	AN_Ca_0600	Barichhola 5	PI 596369	*desi*	BGD	Indian continent	cultivar	USDA
INCCP_00470	10.18730/ZA46R	AN_Ca_0604	W6 19245	W6 19245	*desi*	UZB	Asia	domesticated material	USDA
INCCP_00480	10.18730/ZA4CY	AN_Ca_0614	Floreshtskiy 58/76	W6 19957	*desi*	MDA	Balkanic Europe	landrace	USDA
INCCP_00486	10.18730/Z8V34	AN_Ca_0620	HEERA	PI 612246	*desi*	AUS	others	cultivar	USDA
INCCP_00488	10.18730/ZA4H=	AN_Ca_0622	ILC 72 (differential)	W6 22575	*kabuli*	USA	USA	landrace	USDA
INCCP_00508	10.18730/Z8V89	AN_Ca_0642	ILC 10766	PI 629018	*desi*	SYR	Middle East	breeding material	USDA
INCCP_00509	10.18730/ZA56K	AN_Ca_0643	ARM 172	W6 23903	*desi*	ARM	Caucasus	domesticated material	USDA
INCCP_00512	10.18730/Z8V9A	AN_Ca_0646	CA2969	PI 632396	*kabuli*	ESP	Iberian Peninsula	breeding material	USDA
INCCP_00573	10.18730/ZA703	AN_Ca_0713	ICC 14077	W6 25914	*desi*	IND	Indian continent	landrace	USDA
INCCP_00700	10.18730/ZAAYJ	AN_Ca_0844	G2503	W6 26156	*kabuli*	GEO	Caucasus	landrace	USDA
INCCP_00709	10.18730/ZAB6T	AN_Ca_0853	TJK04:11-067	W6 26223	*kabuli*	TJK	Asia	landrace	USDA
INCCP_00792	10.18730/Z8WWR	AN_Ca_0940	ILC 176	PI 670537	*kabuli*	MAR	Nord Africa	landrace	USDA
INCCP_00853	10.18730/Z8YSB	AN_Ca_1008	ILC 563	PI 670605	*kabuli*	LBN	Middle East	landrace	USDA
INCCP_00918	10.18730/Z90T2	AN_Ca_1074	ILC 1116	PI 670671	*kabuli*	IRN	Middle East	landrace	USDA
INCCP_00955	10.18730/Z91Z2	AN_Ca_1111	ILC 1759	PI 670720	*kabuli*	CHL	South America	landrace	USDA
INCCP_01244	10.18730/Z9B0*	AN_Ca_1408	ILC 4312	PI 671021	*kabuli*	ESP	Iberian Peninsula	landrace	USDA
INCCP_01283	10.18730/Z9C7$	AN_Ca_1447	ILC 4427	PI 671060	*kabuli*	UZB	Asia	landrace	USDA
INCCP_01396	10.18730/Z9FRU	AN_Ca_1561	ILC 4992	PI 671175	*kabuli*	SYR	Middle East	landrace	USDA
INCCP_01429	10.18730/Z9GS*	AN_Ca_1595	ILC 5558	PI 671209	*kabuli*	CYP	Middle East	landrace	USDA

INCCP_01461	10.18730/Z9HSV	AN_Ca_1628	ILC 5791	PI 671242	*kabuli*	TUR	Turkey	landrace	USDA
INCCP_01596	10.18730/Z9P0E	AN_Ca_1767	ILC 6146	PI 671383	*kabuli*	PRT	Iberian Peninsula	landrace	USDA
INCCP_01607	10.18730/Z9PBS	AN_Ca_1778	ILC 6193	PI 671394	*kabuli*	DZA	Nord Africa	landrace	USDA
INCCP_01615	10.18730/Z9PK~	AN_Ca_1786	ILC 6264	PI 671402	*kabuli*	SYR	Middle East	landrace	USDA
INCCP_01867	10.18730/Z9YFT	AN_Ca_2041	ILC 9757	PI 671659	*kabuli*	KAZ	Asia	landrace	USDA
INCCP_01917	10.18730/ZA012	AN_Ca_2091	ILC 11968	PI 671748	*kabuli*	PRT	Iberian Peninsola	landrace	USDA
